# Amplification of Asymmetry via Structural Transitions in Supramolecular Polymer–Surfactant Coassemblies

**DOI:** 10.1021/jacs.5c04047

**Published:** 2025-05-08

**Authors:** Freek V. de Graaf, Christian Zoister, Boris Schade, Tarek Hilal, Xianwen Lou, Stefan Wijker, Sandra M. C. Schoenmakers, Ghislaine Vantomme, Rainer Haag, Abhishek K. Singh, E. W. Meijer

**Affiliations:** † Institute for Complex Molecular Systems, Laboratory of Macromolecular and Organic Chemistry, 3169 Eindhoven University of Technology, PO Box 513, 5600 MB Eindhoven, The Netherlands; ‡ Institute of Chemistry and Biochemistry, Freie Universität Berlin, Takustraβe 3, 14195 Berlin, Germany; § Forschungszentrum für Elektronenmikroskopie und Gerätezentrum BioSupraMol, Institut für Chemie und Biochemie, 9166 Freie Universität Berlin, Fabeckstraße 36a, 14195 Berlin, Germany; ∥ School of Chemistry and RNA Institute, University of New South Wales, Sydney, New South Wales 2052, Australia; ⊥ Max Planck Institute for Polymer Research, Ackermannweg 10, 55128 Mainz, Germany

## Abstract

Asymmetric structures are widespread in nature and essential for life and biointeractive materials. Although nature uniformly operates with homochirality, the hierarchical control of asymmetry in synthetic, water-soluble molecular systems is still underexplored. In this work, we present the amplification of helical asymmetry of benzene-1,3,5-tricarboxamide (BTA) supramolecular polymers by coassembly with homochiral nonionic surfactants. For these mixtures, a strong amplification of asymmetry was observed from the surfactant’s molecular chirality to a preferred helicity of the coassembled polymers. This amplification showed maxima at identical stoichiometric ratios for structurally distinct chiral surfactants, demonstrating the similarity of the coassembly mechanism. Notably, the surfactant-induced asymmetry was completely overridden by the introduction of stereogenic centers into the BTA structure, emphasizing the subtlety of the amplification process. Using a combination of spectroscopy and microscopy, we found that surfactants coassemble with the supramolecular polymers to change fiber morphology from racemic double helices to single helices with a preferred handedness. Furthermore, the coassemblies showed a unique combination of structures and dynamics. Our results elucidate the consequences of supramolecular polymer–surfactant coassembly, offering valuable insights into the resulting asymmetric structures.

## Graphical abstract

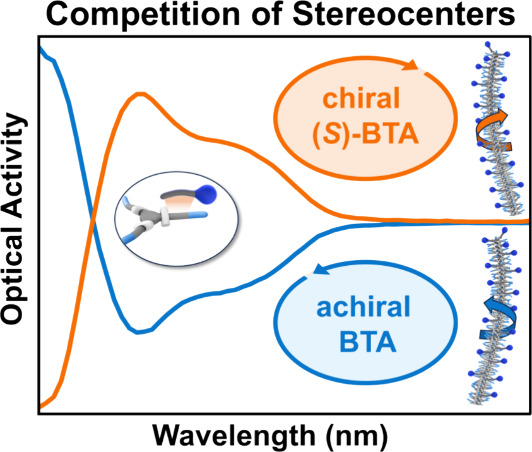

## Introduction

Supramolecular polymers are widely found in biology and used as a basis for synthetic biomaterials and therapeutics.^1–9^ Assembling molecules into functional materials by supramolecular interactions requires fine control over structure, dynamics and chirality.^10^ Particularly, the establishment of homochirality throughout all natural systems remains one of today’s most intriguing research topics.^11–17^ Commonly, the hierarchical amplification of asymmetry in synthetic noncovalent systems goes via additives such as cosolvents and comonomers,^18,19^ which is typically studied with Green’s Sergeant-and-Soldiers and Majority-Rules experiments in organic media.^20,21^ While these studies have provided valuable insights into the mechanisms of asymmetry amplification, examples of this phenomenon in water remain scarce.^22–24^ To truly understand the origins of homochirality, aqueous multicomponent systems that allow for studying these effects are essential.^25–27^

In earlier studies, we have extensively studied the morphology and chirality of benzene-1,3,5-tricarboxamide (BTA)-based helical supramolecular polymers (Scheme 1a). Achiral **nBTA** in water forms a racemic mixture of double helical polymers, similar to crystallization of conglomerates.^25^ The chiral BTAs (*S*)-**D-BTA** and (*S*)-**Me-BTA** (Scheme 1a) have been synthesized to create supramolecular polymers with a helical excess. The (*S*)-**D-BTA**, with a hydrogen–deuterium substitution on the first methylene of all three alkyl tails, introduces a (*S*)-stereocenter. Consequently, the assemblies express weak circular dichroism (CD) that increases with time.^28^ The (*S*)-**Me-BTA**s have (*S*)-configured methyl substituents on the third methylene in each alkyl tail, resulting in assemblies that express high CD intensity. These differences in CD result from distinct fiber morphologies: (*S*)-**D-BTA**, similar to **nBTA**, forms double helices, whereas (*S*)-**Me-BTA** forms single helices. Thus, small changes in molecular structure have a profound effect on supramolecular asymmetry and morphology, but typically require extensive synthetic efforts with uncertain results.

**Scheme 1:**
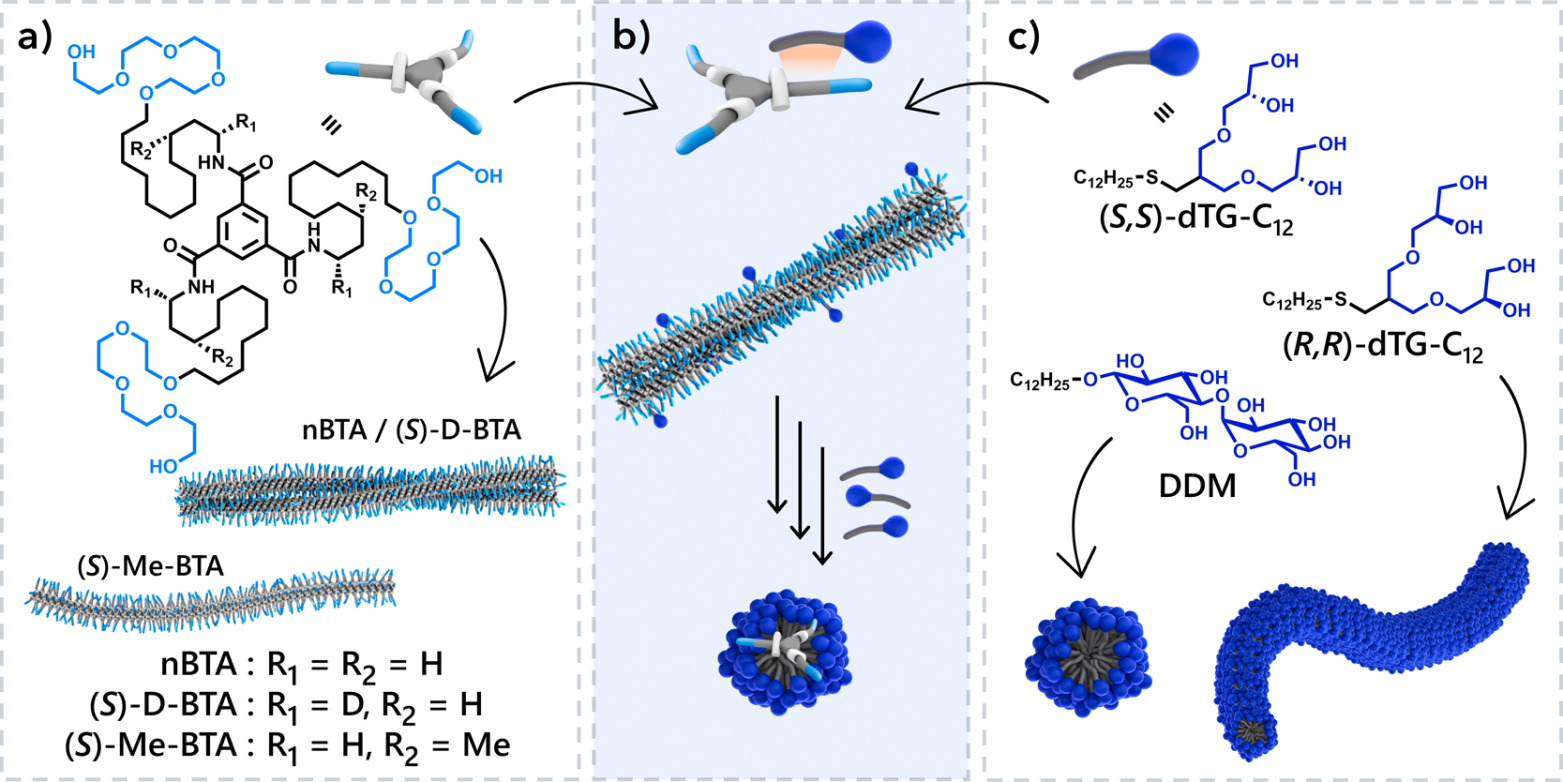
Structures of BTAs Used in This Study^a^

a (a): achiral **nBTA** and chiral (*S*)-**D-BTA**, self-assembling into double helices, and chiral (*S*)-**Me-BTA** forming single helices. (b) Coassembly of **nBTA** with chiral surfactants through hydrophobic interactions, intercalating in the double helical polymers for limited surfactant equivalents. High surfactant equivalents results in the transition into **nBTA**/surfactant comicelles. (c) Structures of (*S,S*)- or (*R,R*)-**dTG-C**_**12**_ and **DDM**, which assemble into wormlike and spherical micelles, respectively.

Surfactants as molecular additives offer an attractive alternative to modulate supramolecular polymers in water. Their amphiphilic nature, which linearly combines hydrophobic and hydrophilic segments, allows for great structural diversity and easy synthetic access.^29,30^ Recent work has shown that supramolecular polymer–surfactant mixtures can result in complex phase-separated domains and sequential gelation transitions upon dilution.^31,32^ It has been shown that surfactants can disrupt the network formed by entangled supramolecular polymers by disassembling the BTA double helices, yielding monomer-surfactant comicelles. It is proposed that this interaction occurs the intercalation of the surfactant's alkyl tail into the polymer's hydrophobic interior (Scheme 1b). However, a general understanding of these interactions is lacking, hampering the potential of surfactants to control the properties of supramolecular assemblies in water.

In this work, we present our discovery that a strong amplification of asymmetry is observed by polymer–surfactant coassembly using achiral **nBTA**-based supramolecular polymers and two structurally distinct chiral nonionic surfactants: **dTG-C**_**12**_ and dodecyl-β-D-maltoside (**DDM**) (Scheme 1c). In addition, we investigated the competition between the chirality of the surfactant and the chiral BTAs, showing the energetic subtlety by which hierarchical amplification occurs . Finally, we were able to relate the induced asymmetry to structural transitions in the **nBTA**-surfactant coassemblies from double to single helices as a function of stoichiometry.

## Results and Discussion

### Asymmetry in Mixtures of nBTA and Chiral Surfactants

The two enantiomers of the single tail amphiphile **dTG-C_12_** were synthesized from enantiomer pure solketal using thiol-ene click chemistry (Scheme S1). **DDM** was purchased and used as received. In water, (*S,S*)- and (*R,R*)-**dTG-C**_**12**_ form worm-like micelles above their critical micellar concentration (CMC) of 0.7 mM (Figure S1). **DDM** is forming spherical micelles above its CMC of 0.17 mM specified by the vendor. The surfactants were mixed in different stoichiometric ratios with the preformed racemic double helices of nBTA following the protocol as specified in the Supporting Information. Figure 1a,b show the CD and UV–vis absorption spectra for mixtures of **nBTA** with (*S,S*)-**dTG-C**_**12**_ and **DDM**, respectively, after 1 week of equilibration. The absence of chromophores in the **dTG-C**_**12**_ and **DDM** structures ensures that the UV–vis absorption and optical activity in the monitored region of 190–350 nm are related only to the molecular organization of **nBTA** (Figure S2). When 0.8 mol equiv of surfactant was exceeded with respect to 0.5 mM **nBTA**, a new UV–vis absorption spectrum appeared, characterized by a single maximum at 193 nm. Based on our previous work,^32^ we hypothesized that this spectrum results from concentration-dependent polymer–surfactant coassembly through hydrophobic interactions, possibly resulting in comicelles (Scheme 1b). Indeed, highly diluted samples (20- to 40-fold) showed UV–vis absorption transitions toward the original **nBTA** absorption spectrum (Figures S3–S5), indicating dilution-induced reassembly of the original double helices.

**Figure 1:**
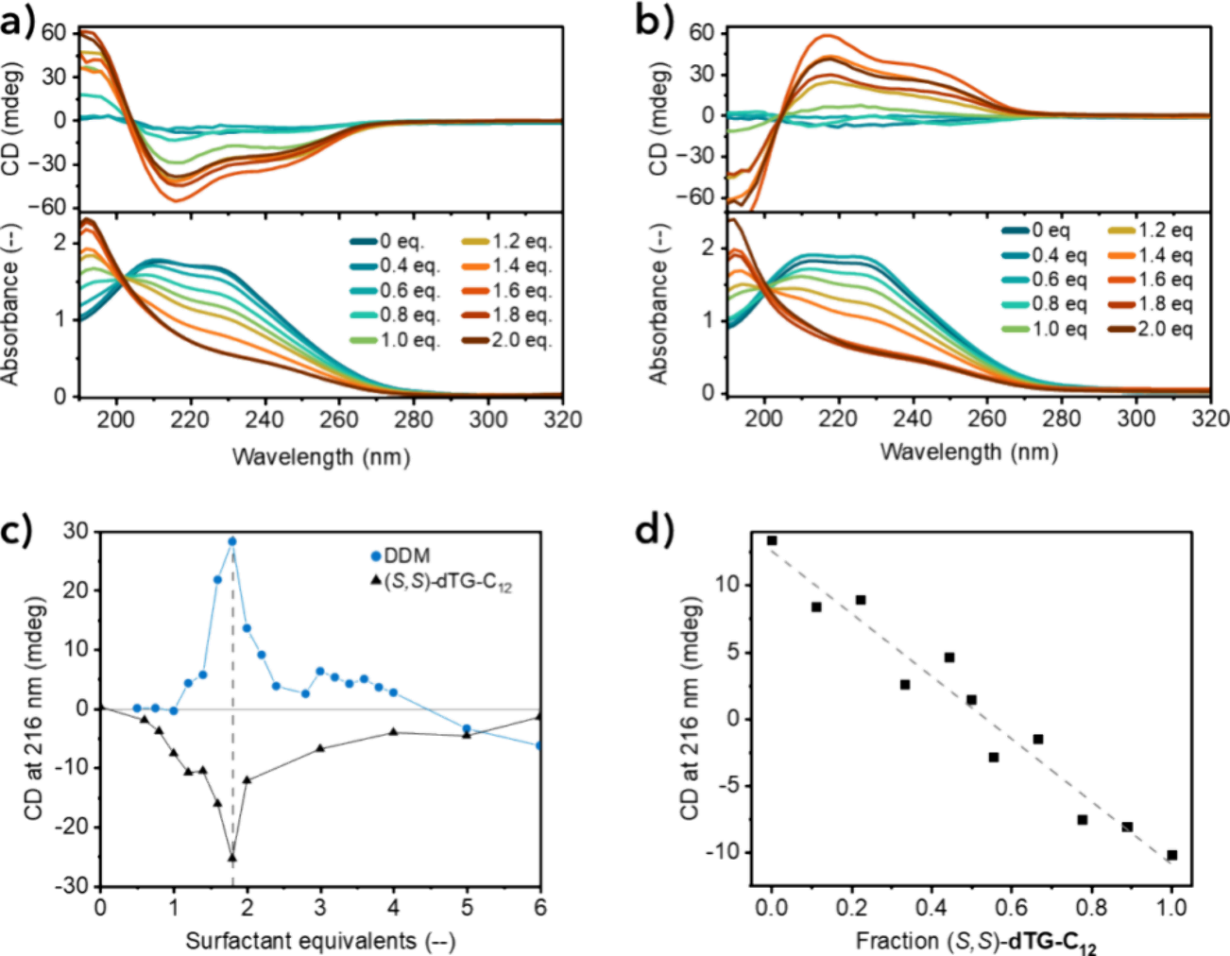
CD and UV–vis absorption spectra of **nBTA** (0.5 mM) mixed with 0–2.0 equiv of (a) (*S*,*S*)*-* **dTG-C**_**12**_ and (b) **DDM** in water. (c) CD at 216 nm for mixtures of **nBTA** (0.25 mM) with 0–6.0 equiv of (*S,S*)-**dTG-C**_**12**_ (black triangles) and **DDM** (blue dots). The vertical dashed line indicates 1.8 equiv of surfactant. (d) CD at 216 nm for mixtures of **nBTA** (0.25 mM) with 1.5 equiv of a (*S*,*S*)-/(*R*,*R*)-**dTG-C**_**12**_ ratio ranging from 0/1 to 1/0.

Interestingly, (*S,S*)-**dTG-C**_**12**_ and **DDM** induced optical activity in mixtures with **nBTA** in an identical manner, with maximum intensities observed at 1.8 mol equiv. Higher equivalents resulted in decreased CD intensities (Figure 1c). Mixing (*R,R*)-**dTG-C**_**12**_ with **nBTA** resulted the mirror image CD of the mixture with (*S,S*)-**dTG-C**_**12**_, indicating that the molecular chirality of the surfactant was translated into a preferred helicity (asymmetry) of **nBTA**-based polymers (Figure S6a). We also varied the molar ratio of (*S,S*)-/(*R,R*)-**dTG-C**_**12**_ at a constant 1.5 equiv to **nBTA** (0.25 mM) and found that the optical activity was linearly correlated with the (*S,S*)-/(*R,R*)-**dTG-C**_**12**_ ratio (Figures 1d and S6b). Thus, no Majority-Rules effect was at play in the coassembled system.

Comparable examples of amplification of asymmetry in water-based supramolecular polymer systems remain scarce, in particular studies that employ Sergeant-and-Soldiers or Majority-Rules principles.^33,34^ A limited number of reports have demonstrated chirality transfer from chiral molecular additives, such as amino acids or surfactants, to racemic supramolecular polymers.^35–39^ These reports typically rely on ionic interactions between the monomer and additive, with the stoichiometric ratio playing a critical role in the degree of asymmetry, consistent with our observations. To the best of our knowledge, no reports have described the transfer of chiral information from nonionic chiral surfactants to nonionic racemic supramolecular polymers in water. More intriguingly, our findings, in combination with the reported examples, raise the question of how overall asymmetry emerges from a competition between the chirality of the surfactant and that of the monomer.

### Asymmetry in Mixtures of Chiral BTAs and Chiral Surfactants

To understand the optical activity observed in the coassemblies of achiral **nBTA** and our chiral surfactants, their absorption and CD spectra were compared with those of previously published chiral BTAs: (*S*)-**D-BTA** and (*S*)-**Me-BTA**. Striking similarities were observed depending on the ratio of **nBTA** to chiral surfactant (Figure 2a). At low surfactant ratios, the CD spectra showed two maxima at 212 and 250 nm, while the UV–vis absorption spectrum remained unchanged, consistent with the UV–vis and CD spectra of (*S*)-**D-BTA** double helices. At surfactant ratios >1.0 mol equiv, the CD spectrum shifted to a maximum at 216 nm with increased intensity, accompanied by a new UV–vis absorption single maximum at 193 nm. These spectra were very similar to those of (*S*)-**Me-BTA** single helices. Therefore, the similarities suggested that low chiral surfactant ratios with achiral nBTA polymers result in asymmetric double helices similar to (*S*)-**D-BTA**, while higher surfactant ratios result in asymmetric single helices similar to those formed by (*S*)-**Me-BTA**.

**Figure 2:**
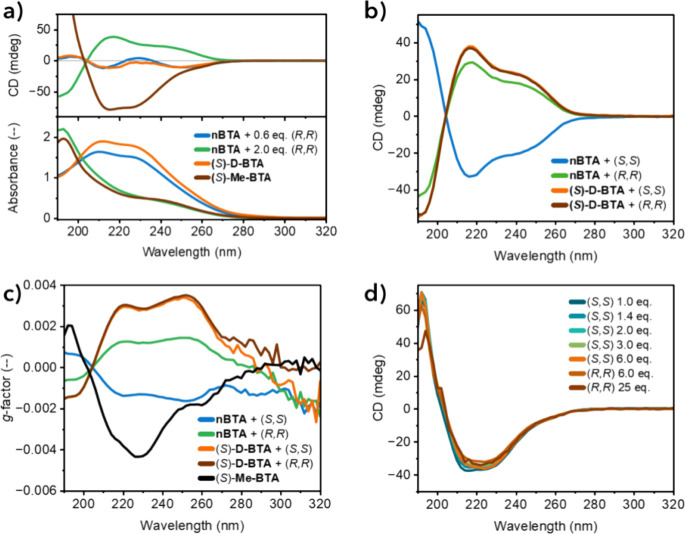
(a) Collection of CD and UV–vis absorption spectra of mixtures of **nBTA** (0.5 mM) with 0.6 (blue) and 2.0 (green) equivalents of (*R,R*)-**dTG-C**_**12**_ and solutions of pure (*S*)-**D-BTA** (0.5 mM, orange) and (*S*)-**Me-BTA** (0.5 mM, brown). (b) CD spectra of **nBTA** (0.5 mM) and (*S*)-**D-BTA** (0.25 mM) mixed with 1.8 mol equiv of (*S,S*)- (blue and orange) and (*R,R*)-**dTG-C**_**12**_ (green and brown). (c) *g*-factors calculated from the CD spectra in b. The *g*-factor of pure (*S*)-**Me-BTA** in water (0.25 mM, black) is added for comparison. (d) CD spectra of (*S*)-**Me-BTA** (0.25 mM) mixed with various equivalents of (*S,S*)- or (*R,R*)-**dTG-C**_**12**_.

Having established these similarities, the asymmetry resulting from a competition between stereocenters on the surfactant versus on the BTA was investigated. Instead of the linear correlation observed for various ratios of (*S,S*)-/(*R,R*)**-dTG-C**_**12**_ mixed with **nBTA**, we consistently found strong positive Cotton effects when mixed with (S)-**D-BTA** (Figure 2b). The CD intensity peaked at 1.8 equiv of chiral surfactant with a UV–vis absorption maximum at 193 nm, suggesting an identical double to single helix transition as seen in mixtures with **nBTA** (Figure S7). However, the helical excess of the resulting single fibers was now completely dominated by the deuterium stereocenters of (*S*)-**D-BTA**. To comment purely on the expression of asymmetry in each of these mixtures, the dissymmetry factor (*g*-factor), which normalizes the CD by absorption, was determined (Figure 2c). These calculated *g-*factors showed that the single helices coassembled from (*S*)-**D-BTA** and **dTG-C**_**12**_ exhibited approximately twice the degree of asymmetry compared to the coassemblies with **nBTA**. Assuming that the maximum achievable asymmetry is defined by the single helices formed by pure (*S*)-**Me-BTA** in water, the mixtures of (*S*)-**D-BTA** with 1.8 equiv of **dTG-C**_**12**_ reach 80% of this degree of asymmetry. Remarkably, in organic solution, a chiral deuterated BTA reaches only one-third of the helical excess observed for its chiral methylated counterpart.^40^ Thus, coassembly of surfactants with the water-compatible deuterated BTA is highly effective in producing single polymers with high helical excess. Mixtures of both **dTG-C**_**12**_ enantiomers with (*S*)-**Me-BTA** single helices were also examined, but showed no changes in CD sign or intensity (Figure 2d). The apparent resilience can be attributed to the absence of a double-to-single helix transition, as (*S*)-**Me-BTA** already forms single helices. Nevertheless, the CD spectra indicate that the degree of asymmetry in the system remains unchanged, though they do not rule out potential surfactant-induced effects on fiber length or dynamics (vide infra).

The spatial proximity of the deuterium and methyl substituents to the BTA core likely explains their dominance in helical excess over chiral surfactants. We expect hydrophobicity to be the sole driving force for coassembly, positioning the surfactant alkyl tail in the hydrophobic region of the supramolecular polymer. Therefore, the chiral hydrophilic groups are oriented toward the aqueous environment, allowing only a subtle induction of helical preference. Nevertheless, it is noteworthy that in the absence of stereogenic information in the design of **nBTA**, these chiral head groups induce a pronounced asymmetry despite their distance from the BTA core. Therefore, the amplification of asymmetry likely results from the cooperativity inherent to the supramolecular polymer, analogous to the preferred handedness adopted by achiral supramolecular polymers in chiral solvents.^18^

### Morphological Transitions of BTA/Surfactant Coassemblies

To further confirm the transition from double to single helices as a result of **nBTA**/surfactant coassembly, we subjected the mixtures to Fourier transform infrared spectroscopy (FTIR) and small-angle X-ray scattering (SAXS). FTIR allowed us to investigate possible changes in the intramolecular hydrogen bonds between amides of adjacent monomers. Reference samples of **nBTA**, (*S*)-**D-BTA** and (*S*)-**Me-BTA** (Figure 3a) were compared against mixtures of **nBTA** and (*S,S*)-**dTG-C**_**12**_ (Figure 3b) and **DDM** (Figure S8). A gradual transition in the absorption profile of the amide I vibration was observed from a single peak for pure **nBTA** to a split profile with increasing molar equivalents of (*S,S*)-**dTG-C**_**12**_ (Figure 3b). A similar observation was made in the mixtures with **DDM** (Figure S8). We attribute the shift in the IR spectrum to a different arrangement in the amide orientation for BTAs arranged in a double or single helix, similar to that shown by Nakano and co-workers for chiral BTAs in organic solvents.^41^ The FTIR spectra before and after the addition of the chiral surfactant showed high similarities to the spectra of (*S*)-**D-BTA** and (*S*)-**Me-BTA**, respectively. Therefore, the FTIR results confirmed that the addition of nonionic chiral surfactants to nBTA shifts the composition from morphologies similar to (*S*)-**D-BTA** (double helices) to those of (*S*)-**Me-BTA** (single helices), presumably via a change in internal packing due to surfactant intercalation.

**Figure 3:**
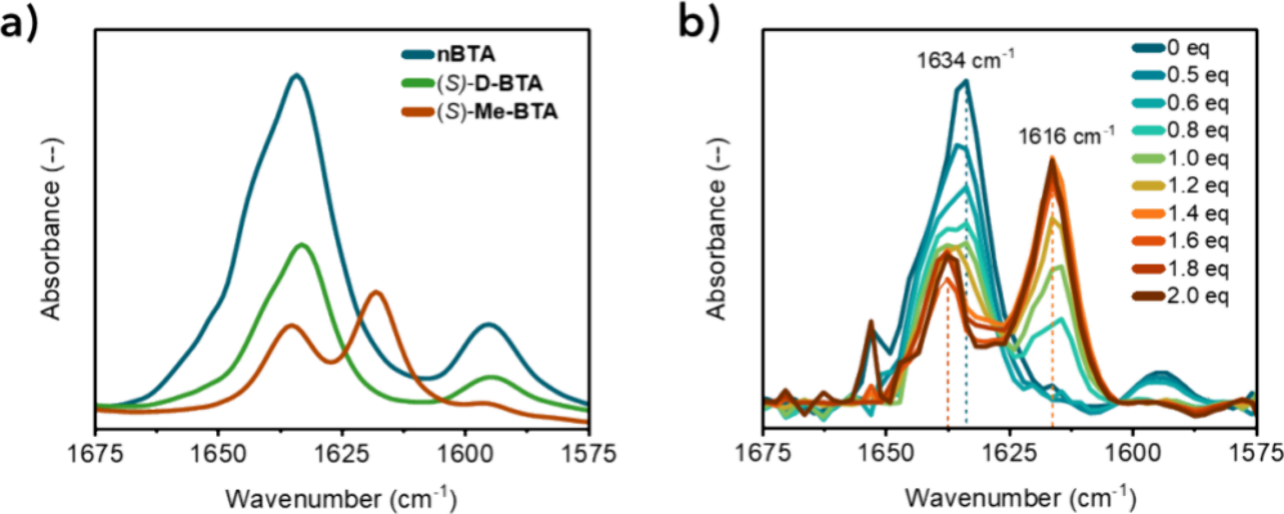
FTIR spectra of the amide I absorption of (a) **nBTA** (blue), (*S*)-**D-BTA** (green) and (*S*)-**Me-BTA** (brown) at 4.0 mM in water. (b) Collection of FTIR spectra for mixtures of **nBTA** (1.0 mM) with 0–2.0 mol equiv of (*S,S*)-**dTG-C**_**12**_.

Structural features of the coassembly were studied using small-angle X-ray scattering (SAXS). Samples of **nBTA**, both chiral surfactants and mixtures of these components were prepared at various concentrations. The reference samples of **nBTA** all showed elongated structures that fit well with a circular cylinder form factor model (Figure 4a). The noise in the high *q* region (>0.1 Å) did not allow us to distinguish between circular and elliptical cylinders. Reference samples of (*R,R*)-**dTG-C**_**12**_ in concentrations ranging from 1.0 to 2.5 mg mL^–1^ (corresponding to 2.3 and 5.7 mM, respectively) all showed assemblies of elongated structures that fit the cylindrical core–shell form factor model (Figure 4b). Reference samples of **DDM** in the same concentration range (corresponding to 2.0 and 4.9 mM, respectively) all fit spherical core–shell structures, i.e. spherical micelles (Figure 4c). The mixtures of **nBTA** with (*R,R*)-**dTG-C**_**12**_ were uninformative for the depiction of coassembled structures, as the individual components both assemble into rod-shaped structures with lengths greater than 60 nm, resulting in scattering profiles that were too much alike (Figures 4d and S9). On the other hand, the scattering data of the mixture of **nBTA** with 4.0 mol equiv of **DDM** revealed the presence of structures that differed from the linear sum of the **nBTA** and **DDM** reference scattering profiles, indicating that the two individual components coassembled into a new elongated structure rather than self-sorted (Figure 4e,f).^42,43^

**Figure 4:**
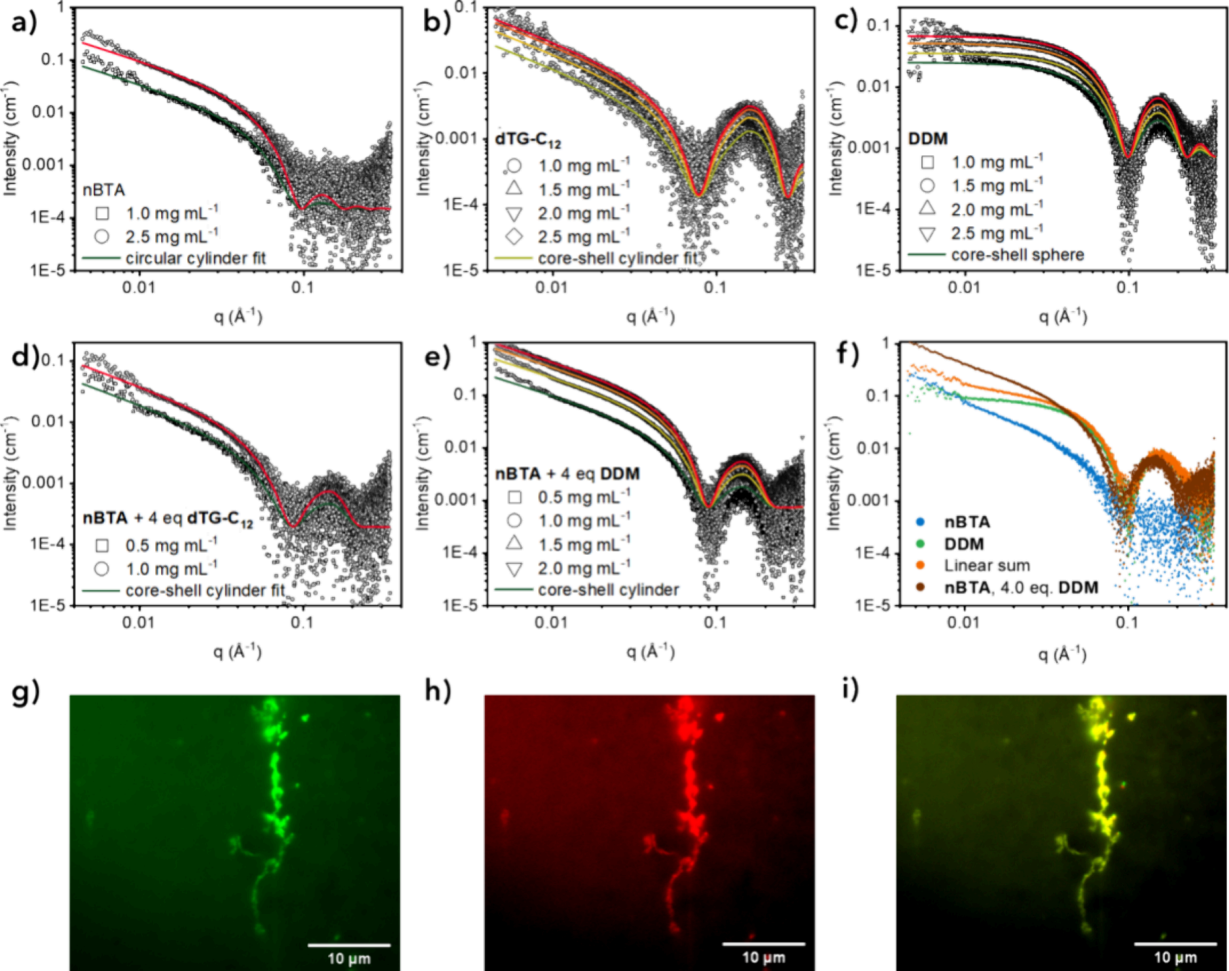
SAXS profiles and fits to corresponding form factor models of (a) **nBTA**, (b) (*S,S*)-**dTG-C**_**12**_ and (c) **DDM** reference samples, and mixtures of **nBTA** with 4.0 mol equiv of (d) (*S,S*)-**dTG-C**_**12**_ and (e) **DDM** in water. For (d) and (e), the concentration corresponds to **nBTA**. (f) Experimental data of **nBTA** (1.0 mg mL^–1^, blue), **DDM** (2.0 mg mL^–1^, green), the calculated linear sum of these two profiles (orange) and the experimental profile of **nBTA** (1.0 mg mL^–1^) with 4.0 mol equiv of **DDM** (corresponding to 1.7 mg mL^–1^). TIRF microscopy images of the (g) Cy3, (h) Cy5 and (i) merged reporter channels of mixtures of **nBTA** (0.05 mM) containing 5 mol % of Cy5-labeled **nBTA** with 1.8 equiv of (*S,S*)-**dTG-C**_**12**_ containing 5 mol % of Cy3-labeled **dTG-C**_**12**_. Scale bar = 10 μm.

To confirm the coassembly of **nBTA** with the **dTG-C_12_** surfactants, we performed total internal reflection fluorescence (TIRF) microscopy on samples containing 5 mol % Cy3-labeled **dTG-C**_**12**_ with 95 mol % (*R,R*)-**dTG-C**_**12**_ and 5 mol % Cy5-labeled **nBTA** with 95 mol % **nBTA**. Figure 4g–i show the fluorescence channels corresponding to Cy3, Cy5 and a merged image of these two channels. The images show that Cy3-labeled **dTG-C**_**12**_ colocalized with Cy5-labeled **nBTA**. Throughout the mixtures, some small structures were visible only in the Cy3 channel, indicating that homoaggregates of **dTG-C_12_** remained (Figure S10).

Taken together, the results strongly suggest that the surfactants do indeed coassemble with **nBTA** and induce a transition from double to single helix morphology as a function of the stoichiometric ratio. To visualize and verify this hypothesis, cryogenic transmission electron microscopy (cryo-TEM) was performed on mixtures of **nBTA** and the chiral surfactants. Figure 5 shows the electron micrographs of **nBTA** mixed with 1.8, 4.0, and 10 mol equiv of (*S,S*)-**dTG-C**_**12**_ (bottom) or **DDM** (top). The **nBTA** reference sample showed few supramolecular polymers in the bulk solution, while a fingerprint-like pattern was observed (Figure 5a). According to Herziger and co-workers,^25^ this fingerprint likely represents a monolayer of **nBTA** assemblies located at the air–water interface. The absence of this pattern in all mixtures with (*S,S*)-**dTG-C**_**12**_ or **DDM** suggests that **nBTA** at the air–water interface was replaced by the surfactants. As a result, dense fiber networks were observed in both mixtures with 1.8 (Figure 5b) and 4.0 (Figure 5c) molar equivalents of surfactant. Fewer fibers and more fiber ends were present at 10 equiv of surfactant (Figure 5d), confirming that higher equivalents of surfactant disrupt the **nBTA** polymers. Micrographs of pure (*S,S*)-**dTG-C**_**12**_ and **DDM** references in water (Figure 5e) showed wormlike and spherical micelles, respectively, both consistent with the SAXS data.

**Figure 5:**
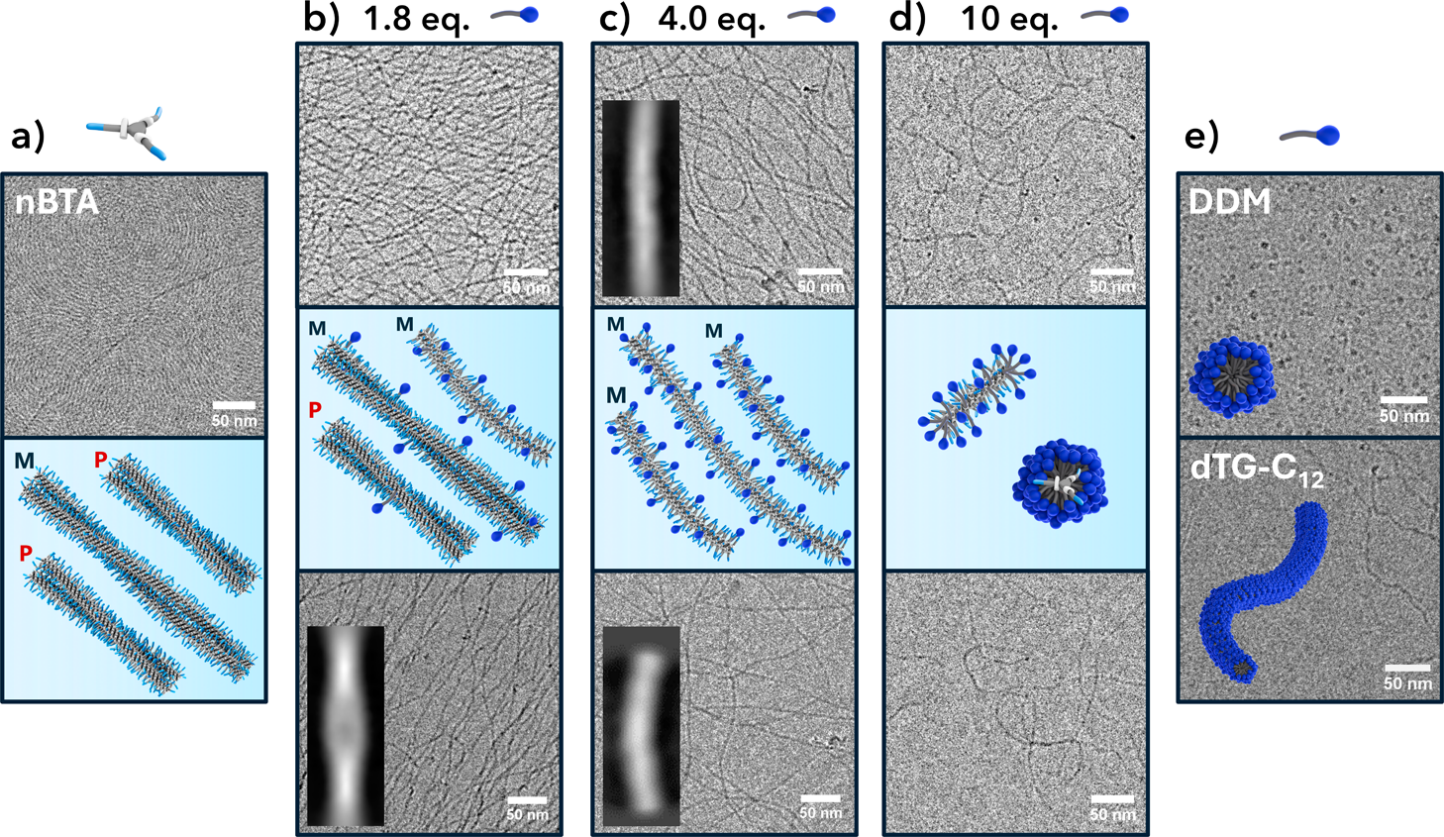
Cryo-TEM micrographs of (left to right): pure **nBTA** (0.25 mM) showing double helices and fingerprint-like patterns. The mixtures with 1.8, 4.0, and 10 mol equiv of **DDM** (top) and (*R,R*)-**dTG-C**_**12**_ (bottom) show fibrous networks which decrease in density for higher surfactant equivalents. Reference micrographs of **DDM** and (*R,R*)-**dTG-C**_**12**_ (2.5 mM) show spherical and wormlike micellar aggregates, respectively. Single particle analysis on individual fibers revealed predominantly double helices in the 1.8 equiv mixtures and single helices in the 4.0 equiv mixtures. The cartoons are added to visualize the asymmetry and morphology of coassemblies in the solutions.

Detailed information at the individual polymer level of the **nBTA**/surfactant coassemblies was obtained by subjecting the micrographs to single particle analysis.^44^ The resulting images are interpreted qualitatively due to noise in the generated images, which may show artificial irregularities along the fiber length. Representative 2D class averages are shown in the insets of the corresponding samples in Figure 5. For samples containing 1.8 equiv of (*S,S*)-**dTG-C**_**12**_, double helices were predominantly observed. For the sample containing 1.8 equiv of **DDM**, the analysis failed due to the high fiber density. Samples containing either 4.0 equiv of (*S,S*)-**dTG-C**_**12**_ or **DDM** showed predominantly single fibers. These findings are in contrast to the spectroscopic results, which showed predominantly single fibers in 1.8 equiv mixtures for both surfactants. We attribute this difference to an estimated 5–10 times higher surface-to-volume ratio of the sample applied to cryo-TEM grids compared to cuvettes, which shifts the distribution of surfactants between the air–water interfaces and the bulk water phase (calculation provided in the Supporting Information). Interestingly, BTA derivatives with identical side chains as the **dTG-C**_**12**_ structure showed very similar morphologies when coassembled with **nBTA**.^25,26^ Therefore, the readily accessible surfactants presented in this study are a powerful tool and valuable alternative in coassembled supramolecular structures, providing fine control over morphology and asymmetry.

### Dynamics of BTA and (Chiral) Surfactant Coassemblies

As a final important aspect of **nBTA**-chiral surfactant coassembled structures, we investigated the effect on supramolecular polymer dynamics. Hydrogen–deuterium exchange followed by mass spectrometry (HDX–MS) experiments on samples of **nBTA** with 0.5 and 1.0 equiv of (*R,R*)-**dTG-C_12_**. Both samples were diluted 100-fold in deuterium oxide (D_2_O) and various deuterated species were monitored over time by mass spectrometry (Figure S11). The species exchanging labile protons on the outer hydroxyl groups (3D) and on the inner amide groups (6D) were followed. We chose (*R,R*)-**dTG-C**_**12**_ over **DDM** for this experiment because the interaction between **nBTA** and (*R,R*)-**dTG-C**_**12**_ was found to be less affected by dilution than the interaction with **DDM** (Figure S3). However, we observed a shift in UV–vis absorption after dilution corresponding to double helices, indicating a decrease in the interaction between **nBTA** and (*R,R*)-**dTG-C**_**12**_ . Nevertheless, the sample containing 0.5 equiv of (*R,R*)-**dTG-C**_**12**_ reached an average of 58% of 6D in the first hour and over 84% of 6D over 72 h (Figure 6a). In comparison, pure **nBTA** typically does not exceed 50% of 6D within the first hour and does not exceed 70% of 6D after 72 h.^45^ Increasing (*R,R*)-**dTG-C**_**12**_ to 1.0 equiv further enhances the dynamics, with a population of 6D of 78% after the first hour and reaching a steady state of 85% after 12 h. Thus, the surfactant intercalation enhances the exchange dynamics for labile hydrogen atoms.

**Figure 6:**
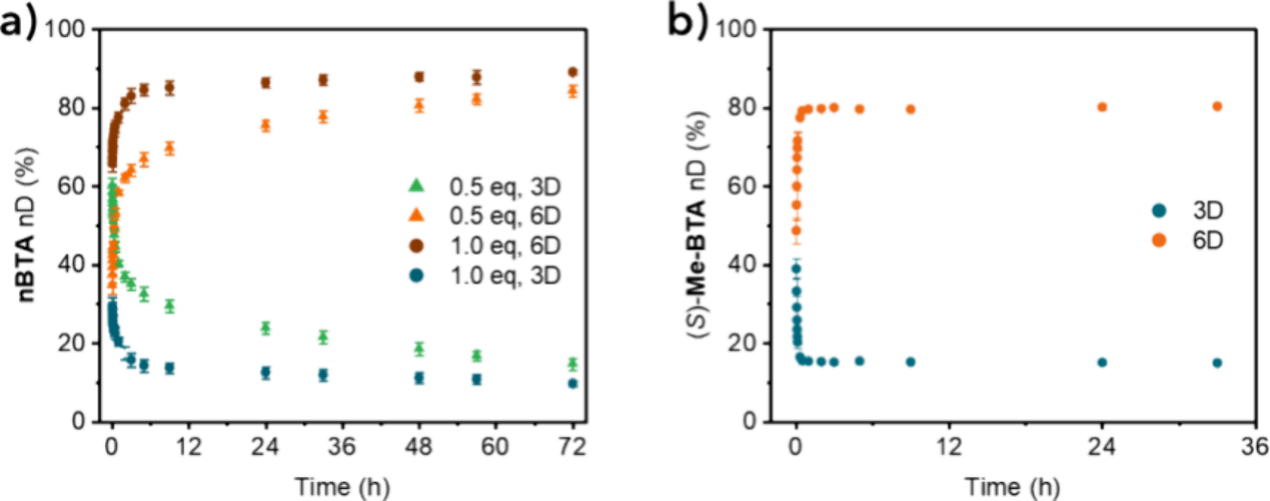
HDX-MS profiles showing 3D and 6D exchanged species of (a) **nBTA** with 0.5 mol equiv (green and orange) and 1.0 mol equiv (brown and blue) and (b) (*S*)-**Me-BTA** with 2.0 mol equiv of (*R,R*)-**dTG-C**_**12**_. The data points were determined by the mean of three identical experiments, with error bars indicating the standard deviation.

Compared to earlier work, where we studied the single helical coassemblies of **nBTA** and **dTG-C_12_** functionalized BTA comonomers,^26^ we observed an opposite trend in dynamics, i.e., the mixture promoting single helices showed higher exchange dynamics. However, the 100-fold dilution required for the HDX-MS experiment does not result in predominantly single helices in solution, as shown by the absorption spectrum. To ensure a fair comparison, the HDX-MS experiments were performed with (*S*)-**Me-BTA** mixed with 2.0 equiv of (*R,R*)-**dTG-C**_**12**_. No evidence of polymer degradation was observed in the data shown in Figure 2d. In addition, pure (*S*)-**Me-BTA** in water exhibits slower exchange dynamics than pure **nBTA** (Figure S11c). In contrast, a mixture of (*S*)-**Me-BTA** with 2.0 mol equiv of (*R,R*)-**dTG-C**_**12**_ showed a 6D population of 80% 1 h after dilution in D_2_O, with no further exchange detectable. Therefore, surfactants greatly enhance the exchange dynamics of BTA monomers, especially in the initial phase of coassembly. These dynamics are a unique feature of **nBTA** and chiral surfactant coassemblies, given that similar morphologies are obtained as for BTA homoassemblies. As the dynamics are crucial for biointeractive applications,^46^ this will have a great impact on potential future applications of BTA and surfactant coassemblies as biomaterials.

## Conclusions

We revealed an unexpected amplification of asymmetry that emerged alongside a sequence of structural transitions in the coassembly of supramolecular polymers with increasing amounts of chiral nonionic surfactants. Under the assumption that only the hydrophobic effect is responsible for the coassembly, the observed strong amplification of asymmetry is remarkable. Moreover, introduction of a stereogenic deuterium atom in the BTA fully overrides the asymmetry effect initially introduced by the chiral surfactant, further confirming the fine energetic balance involved in the surfactant-induced asymmetry. The double-to-single helix transition occurs with an optimal ratio of BTA and chiral surfactant, followed by a decrease in asymmetry as this ratio is further increased. Microscopic data suggest that the coassembled fibers shorten in length and shift toward surfactant-dominated less-organized species, i.e. micelles. The work demonstrates that careful consideration of the supramolecular polymer–surfactant mixing ratio is a powerful tool for controlling structure and asymmetry. Furthermore, our results show that the dynamics of monomer-surfactant coassemblies are completely different from those of monomer homoassemblies. Therefore, these characteristics of asymmetric supramolecular polymer–surfactant coassemblies will result in unique properties when implemented as biomaterials compared to conventional supramolecular polymer systems. Current investigations are ongoing with the application toward virus inhibition, where it is hypothesized that the change in dynamics of the coassembly enables a morphological adaptation to the shape of the virus. Lastly, the findings can help in the understanding of the origin of homochirality, as aqueous system are of greater relevance with respect to systems studying amplification of asymmetry in organic media.

## Supplementary Material



## Notes

Experimental details, synthetic procedures, materials and methods, additional spectroscopic and microscopic data, details on HDX-MS measurements, and ^1^H and ^13^C NMRs (PDF)

## References

[1] Wegner A., Engel J. (1975). Kinetics of the Cooperative Association of Actin to Actin Filament. Biophys. Chem..

[2] Romeiro
Motta M., Biswas S., Schaedel L. (2023). Beyond Uniformity:
Exploring the Heterogeneous and Dynamic Nature of the Microtubule
Lattice. Eur. J. Cell Biol..

[3] Knowles T. P. J., Vendruscolo M., Dobson C. M. (2014). The Amyloid State and Its Association
with Protein Misfolding Diseases. Nat. Rev.
Mol. Cell Biol..

[4] Freeman R., Han M., Álvarez Z., Lewis J. A., Wester J. R., Stephanopoulos N., McClendon M. T., Lynsky C., Godbe J. M., Sangji H., Luijten E., Stupp S. I. (2018). Reversible Self-Assembly
of Superstructured Networks. Science.

[5] Yuan S. C., Lewis J. A., Sai H., Weigand S. J., Palmer L. C., Stupp S. I. (2022). Peptide Sequence
Determines Structural Sensitivity
to Supramolecular Polymerization Pathways and Bioactivity. J. Am. Chem. Soc..

[6] Rutten M. G. T. A., Rijns L., Dankers P. Y. W. (2024). Controlled,
Supramolecular Polymer
Formulation to Engineer Hydrogels with Tunable Mechanical and Dynamic
Properties. J. Polym. Sci..

[7] Howlett M. G., Scanes R. J. H., Fletcher S. P. (2021). Selection
between Competing Self-Reproducing
Lipids: Succession and Dynamic Activation. JACS
Au.

[8] Frederix P. W. J. M., Scott G. G., Abul-Haija Y. M., Kalafatovic D., Pappas C. G., Javid N., Hunt N. T., Ulijn R. V., Tuttle T. (2015). Exploring the Sequence Space for (Tri-)­Peptide Self-Assembly
to Design and Discover New Hydrogels. Nat. Chem..

[9] Ghanbari E., Picken S. J., van Esch J. H. (2023). Design
Rules for Binary Bisamide
Gelators: Toward Gels with Tailor-Made Structures and Properties. Langmuir.

[10] Shao L., Ma J., Prelesnik J. L., Zhou Y., Nguyen M., Zhao M., Jenekhe S. A., Kalinin S. V., Ferguson A. L., Pfaendtner J., Mundy C. J., De Yoreo J. J., Baneyx F., Chen C. L. (2022). Hierarchical
Materials from High Information Content Macromolecular Building Blocks:
Construction, Dynamic Interventions, and Prediction. Chem. Rev..

[11] Yang Z., Jaiswal A., Yin Q., Lin X., Liu L., Li J., Liu X., Xu Z., Li J. J., Yong K. T. (2024). Chiral
Nanomaterials in Tissue Engineering. Nanoscale.

[12] Morrow S.
M., Bissette A. J., Fletcher S. P. (2017). Transmission of Chirality through
Space and across Length Scales. Nat. Nanotechnol..

[13] Sato K., Ji W., Álvarez Z., Palmer L. C., Stupp S. I. (2019). Chiral
Recognition of Lipid Bilayer Membranes by Supramolecular Assemblies
of Peptide Amphiphiles. ACS Biomater. Sci. Eng..

[14] Blackmond D. G. (2019). The Origin
of Biological Homochirality. Cold Spring Harb.
Perspect. Biol..

[15] Deng M., Yu J., Blackmond D. G. (2024). Symmetry
Breaking and Chiral Amplification in Prebiotic
Ligation Reactions. Nature.

[16] Yang S., Geiger Y., Geerts M., Eleveld M. J., Kiani A., Otto S. (2023). Enantioselective Self-Replicators. J. Am. Chem.
Soc..

[17] Klawa S. J., Lee M., Riker K. D., Jian T., Wang Q., Gao Y., Daly M. L., Bhonge S., Childers W. S., Omosun T. O., Mehta A. K., Lynn D. G., Freeman R. (2024). Uncovering Supramolecular
Chirality Codes for the Design of Tunable Biomaterials. Nat. Commun..

[18] Ślęczkowski M. L., Mabesoone M. F. J., Ślęczkowski P., Palmans A. R. A., Meijer E. W. (2021). Competition
between Chiral Solvents and Chiral Monomers
in the Helical Bias of Supramolecular Polymers. Nat. Chem..

[19] Kim T., Mori T., Aida T., Miyajima D. (2016). Dynamic Propeller Conformation
for the Unprecedentedly High Degree of Chiral Amplification of Supramolecular
Helices. Chem. Sci..

[20] Green M., Reidy M. (1989). Macromolecular
Stereochemistry: The out-of-Proportion
Influence of Optically Active Comonomers on the Conformational Characteristics
of Polyisocyanates. The Sergeants and Soldiers Experiment. J. Am. Chem. Soc..

[21] Das A., Ghosh S., George S. J. (2025). Amplification and Attenuation of
Asymmetry via Kinetically Controlled Seed-Induced Supramolecular Polymerization. Angew. Chem., Int. Ed..

[22] Song C. E., Park S. J., Hwang I. S., Jung M. J., Shim S. Y., Bae H. Y., Jung J. Y. (2019). Hydrophobic Chirality Amplification
in Confined Water Cages. Nat. Commun..

[23] Lei Y., Chen Q., Liu P., Wang L., Wang H., Li B., Lu X., Chen Z., Pan Y., Huang F., Li H. (2021). Molecular
Cages Self-Assembled by Imine Condensation in Water. Angew. Chem., Int. Ed..

[24] Karunakaran S. C., Cafferty B. J., Weigert-Muñoz A., Schuster G. B., Hud N. V. (2019). Spontaneous Symmetry Breaking in the Formation of Supramolecular
Polymers: Implications for the Origin of Biological Homochirality. Angew. Chem., Int. Ed..

[25] Lafleur R. P. M., Herziger S., Schoenmakers S. M. C., Keizer A. D. A., Jahzerah J., Thota B. N. S., Su L., Bomans P. H. H., Sommerdijk N. A. J. M., Palmans A. R. A., Haag R., Friedrich H., Böttcher C., Meijer E. W. (2020). Supramolecular Double Helices from
Small C3-Symmetrical Molecules Aggregated in Water. J. Am. Chem. Soc..

[26] Thota B. N. S., Lou X., Bochicchio D., Paffen T. F. E., Lafleur R. P. M., van Dongen J. L. J., Ehrmann S., Haag R., Pavan G. M., Palmans A. R. A., Meijer E. W. (2018). Supramolecular Copolymerization
as a Strategy to Control the Stability of Self-Assembled Nanofibers. Angew. Chem. - Int. Ed.

[27] Xu F., Crespi S., Pacella G., Fu Y., Stuart M. C. A., Zhang Q., Portale G., Feringa B. L. (2022). Dynamic
Control
of a Multistate Chiral Supramolecular Polymer in Water. J. Am. Chem. Soc..

[28] Schoenmakers S. M. C., Spiering A. J. H., Herziger S., Böttcher C., Haag R., Palmans A. R. A., Meijer E. W. (2022). Structure
and Dynamics
of Supramolecular Polymers: Wait and See. ACS
Macro Lett..

[29] Lombardo D., Kiselev M. A., Magazù S., Calandra P. (2015). Amphiphiles Self-Assembly:
Basic Concepts and Future Perspectives of Supramolecular Approaches. Adv. Condens. Matter Phys..

[30] Rashmi R., Hasheminejad H., Herziger S., Mirzaalipour A., Singh A. K., Netz R. R., Böttcher C., Makki H., Sharma S. K., Haag R. (2022). Supramolecular Engineering
of Alkylated, Fluorinated, and Mixed Amphiphiles. Macromol. Rapid Commun..

[31] Duijs H., Kumar M., Dhiman S., Su L. (2024). Harnessing Competitive
Interactions to Regulate Supramolecular “Micelle-Droplet-Fiber”
Transition and Reversibility in Water. J. Am.
Chem. Soc..

[32] Su L., Mosquera J., Mabesoone M. F. J., Schoenmakers S. M. C., Muller C., Vleugels M. E. J., Dhiman S., Wijker S., Palmans A. R. A., Meijer E. W. (2022). Dilution-Induced Gel-Sol-Gel-Sol
Transitions by Competitive Supramolecular Pathways in Water. Science.

[33] Brunsveld L., Lohmeijer B. G. G., Vekemans J. A. J. M., Meijer E. W. (2000). Chiral Amplification
in Dynamic Helical Columns in Water. Chem. Commun..

[34] Yang X., Lu H., Tao Y., Zhang H., Wang H. (2022). Controlling Supramolecular
Filament Chirality of Hydrogel by Co-Assembly of Enantiomeric Aromatic
Peptides. J. Nanobiotechnol..

[35] Rananaware A., La D. D., Al Kobaisi M., Bhosale R. S., Bhosale S. V. (2016). Controlled Chiral Supramolecular
Assemblies of Water Soluble Achiral
Porphyrins Induced by Chiral Counterions. Chem.
Commun..

[36] Franke D., Vos M., Antonietti M., Sommerdijk N. A. J. M., Faul C. F. J. (2006). Induced Supramolecular
Chirality in Nanostructured Materials: Ionic Self-Assembly of Perylene-Chiral
Surfactant Complexes. Chem. Mater..

[37] Dou X., Mehwish N., Zhao C., Liu J., Xing C., Feng C. (2020). Supramolecular Hydrogels with Tunable
Chirality for Promising Biomedical
Applications. Acc. Chem. Res..

[38] Ryu N., Okazaki Y., Hirai K., Takafuji M., Nagaoka S., Pouget E., Ihara H., Oda R. (2016). Memorized Chiral Arrangement
of Gemini Surfactant Assemblies in Nanometric Hybrid Organic-Silica
Helices. Chem. Commun..

[39] Xu H., Lu H., Zhang Q., Chen M., Shan Y., Xu T. Y., Tong F., Qu D. H. (2022). Surfactant-Induced Chirality Transfer,
Amplification and Inversion in a Cucurbit[8]­Uril-Viologen Host-Guest
Supramolecular System. J. Mater. Chem. C.

[40] Cantekin S., Balkenende D. W. R., Smulders M. M. J., Palmans A. R. A., Meijer E. W. (2011). The Effect
of Isotopic Substitution on the Chirality of a Self-Assembled Helix. Nat. Chem..

[41] Nakano Y., Markvoort A. J., Cantekin S., Filot I. A. W., Ten
Eikelder H. M. M., Meijer E. W., Palmans A. R. A. (2013). Conformational
Analysis of Chiral Supramolecular Aggregates: Modeling the Subtle
Difference between Hydrogen and Deuterium. J.
Am. Chem. Soc..

[42] Spalla, O. General Theorems in Small-Angle Scattering. In Neutrons, X-rays and Light: Scattering Methods Applied to Soft Condensed Matter; Lindner, P. , Zemb, T. , Eds.; Elsevier, 1988.

[43] Kikhney A.
G., Svergun D. I. A. (2015). A Practical
Guide to Small Angle X-Ray Scattering (SAXS)
of Flexible and Intrinsically Disordered Proteins. FEBS Lett..

[44] Frank, J. Three-Dimensional Electron Microscopy of Macromolecular Assemblies: Visualization of Biological Molecules in Their Native State, 1st ed.; Oxford University Press, 1996.

[45] Lou X., Schoenmakers S. M. C., van Dongen J. L. J., Garcia-Iglesias M., Casellas N. M., Fernández-Castaño
Romera M., Sijbesma R. P., Meijer E. W., Palmans A. R. A. (2021). Elucidating
Dynamic
Behavior of Synthetic Supramolecular Polymers in Water by Hydrogen/Deuterium
Exchange Mass Spectrometry. J. Polym. Sci..

[46] Roy N., Schädler V., Lehn J. M. (2024). Supramolecular Polymers: Inherently
Dynamic Materials. Acc. Chem. Res..

