# Supplementary material for: Amplification of Asymmetry via Structural Transitions in Supramolecular Polymer–Surfactant Coassemblies
Source: J Am Chem Soc. 2025 May 8;147(20):17468–76. doi: 10.1021/jacs.5c04047 (PMC12100644; doi:10.1021/jacs.5c04047)
Supplement: Supplementary file 1 [file ja5c04047_si_001.pdf]

Supporting Information for

Amplification of asymmetry via structural transitions in supramolecular polymer-surfactant coassemblies

Freek V. de Graaf,[‡,a] Christian Zoister,[‡,a,b] Boris Schade,[c] Tarek Hilal,[c] Xianwen Lou,[a] Stefan Wijker,[a] Sandra M. C. Schoenmakers,[a] Ghislaine Vantomme,[a] Rainer Haag,[b]* Abhishek K. Singh,[b]* and E.W. Meijer [a,d,e]*.

a Institute for Complex Molecular Systems, Laboratory of Macromolecular and Organic Chemistry, Eindhoven University of Technology, PO Box 513, 5600 MB Eindhoven, The Netherlands.

b Freie Universität Berlin, Institute of Chemistry and Biochemistry, Takustrasse 3, 14195 Berlin, Germany

c Forschungszentrum für Elektronenmikroskopie und Gerätezentrum BioSupraMol, Institut für Chemie und Biochemie, Freie Universität Berlin, Fabeckstraße 36a, 14195 Berlin, Germany.

d School of Chemistry and RNA Institute, University of New South Wales, NSW 2052 Sydney, Australia.

e Max Planck Institute for Polymer Research, Ackermannweg 10, 55128 Mainz, Germany.

‡ both authors contributed equally to this work

* corresponding authors: haag@zedat.fu-berlin.de; abhikmc@zedat.fu-berlin.de; e.w.meijer@tue.nl

Table of Contents

1. Materials and Methods 2

2. Synthesis 3

3. Physicochemical Characterization of Surfactants 8

3.1 Critical micelle concentration of dTG-C_{12} 8

3.2 Spectroscopic data of dTG-C_{12} and DDM 9

4. Coassembly studies 10

4.1 Spectroscopic data of nBTA-surfactant coassemblies 10

4.1.1 Dilution studies 10

4.1.2 Concentration dependent coassembly of **nBTA** with **dTG-C_{12}** and **DDM** 11

4.1.3 (*R*,*R*)-**dTG-C_{12}** and **nBTA** coassembly 13

4.2 Relation of stereoisomers to BTA-fiber asymmetry 13

4.3 Fourier-transform infrared (FTIR) spectroscopy 14

4.4 Small-angle X-ray scattering (SAXS) 15

4.5 Total internal reflection fluorescence (TIRF) microscopy 16

4.6 Hydrogen-deuterium exchange – mass spectrometry (HDX-MS) 17

4.7 Cryo-TEM/Class sum images 18

5. References 20

6. Appendix 21

6.1 NMR 21

1. Materials and Methods

Reagents

All solvents were used in HPLC quality, as well as deuterated solvents. Unless mentioned differently, all reagents and solvents were used as purchased from commercial suppliers, without any prior purification. All reactions which involved air- or water-sensitive compounds were carried out in a dried flask under an argon atmosphere. **nBTA**[1], **(*S*)-D-BTA**[2], **(*S*)-Me-BTA**[1] and **BTA-Cy5**[3] were synthesized following literature procedure.

Thin layer chromatography

Thin layer chromatography (TLC) analysis was performed on Merck silica gel 60, Fluorescenceindikator F_{254}. A solution of KMnO4 was used as a staining solution. The solution contains 1.5 g of $KMnO_4$, 10 g K_2CO_3, and 1.25 mL 10% NaOH in 200mL water.

Column chromatography

The preparative purification of mixtures by column chromatography was conducted on Merck silica gel 60 (0.040–0.063 mm). The different eluents used are described in the synthesis section.

Nuclear Magnetic Resonance (NMR) spectroscopy

^{1}H and ^{13}C NMR spectra were recorded on Bruker Ultrashield 400 MHz spectrometers (400 MHz for ^{1}H NMR; 100 MHz for ^{13}C NMR) or JEOL Spectrometer ECP500 (500 MHz for ^{1}H NMR, 151 MHz for ^{13}C NMR) or JEOL Spectrometer ECZ600 (600 MHz for ^{1}H NMR, 151 MHz for ^{13}C NMR) or Bruker AVANCE700 (700 MHz for ^{1}H NMR, 176 MHz for ^{13}C NMR). Chemical shifts δ are given in ppm relative to TMS as an internal standard or relative to the resonance of the solvent (^{1}H NMR: Chloroform: δ = 7.26 ppm; D_2O: δ = 4.79 ppm; MeOD δ = 3.31 ppm; ^{13}C NMR: Chloroform: δ = 77.00 ppm; MeOD: δ = 49.00 ppm). Deuterated solvents used are indicated in each case. Peak multiplicities are abbreviated as s: singlet; d: doublet; m: multiplet; t: triplet.

Critical micelle concentration (CMC)

The critical micelle concentration was determined by the fluorescence technique using the encapsulation of 'Nile red' as a model dye. A stock solution of the dye in THF (1 mg/mL) was prepared. Each 20 µL of this stock solution were added to 10 sample vials where the THF was allowed to evaporate to leave a thin film of the dye. 5 mg/mL stock solutions of the amphiphiles

in Milli-Q water were stirred overnight. The dilution series of the amphiphile were then transferred to the dye loaded vials and kept stirring overnight. The non-encapsulated dye was removed by filtration through 0.45 μm PTFE filter. Fluorescence measurements were performed using a Cary Eclipse fluorescence spectrophotometer. The determination of the CMC was performed by plotting the fluorescence intensity at $\lambda = 635$ nm against the logarithm of the amphiphile concentration.

Sample preparation

All BTA and surfactant stock solutions were prepared by weighing a certain amount of the dry material in a glass vial using a microgram balance. The desired amount of Milli-Q water, which was purified using an EMD Millipore Milli-Q Integral Water Purification System, was added to the dry material. For **nBTA, (*S*)-D-BTA** and **(*S*)-Me-BTA** stock solutions, solutions were vortexed, heated up to 80°C for 15 minutes while stirring and stirred overnight at room temperature. The final samples containing mixtures of BTA and surfactant were prepared from these stock solutions and treated with the same heating and stirring protocol. Samples were equilibrated at least 1 day prior to measurement.

Circular dichroism (CD), Linear dichroism (LD), Ultraviolet absorption spectroscopy (UV)

Circular dichroism (CD), linear dichroism (LD) and UV absorption spectra were measured on a JASCO J-815 CD spectrometer equilibrated at 20 °C. The measurements were performed in Quartz cuvettes (Hellma) with a pathlength of 10 mm (50 μM), 1 mm (250-750 μM), 0.2 mm (1 mM) or 0.1 mm (2.5 mM). Standard sensitivity, a D.I.T of 2 seconds, a bandwidth of 1.0 nm, a data pitch of 2 nm and a scanning speed of 50 nm/min were used. The spectra were recorded from 350 nm to 190 nm. The spectra were corrected using a baseline of the corresponding solvent.

Fourier transform infrared spectroscopy (FT-IR)

Fourier transform infrared spectroscopy (FT-IR) spectra were recorded on a Perkin Elmer Spectrum Two FT-IR spectrometer. Liquid samples were measured at room temperature from 1800 cm^{-1} to 1500 cm^{-1} over 16 scans in a BaF_2 cell with a spacer of 0.06 mm.

Small-angle x-ray scattering (SAXS)

SAXS was performed at the Diamond beamline B21 – BioSAXS (Diamond Light Source Ltd., Didcot, United Kingdom, SM-31952).[4] The scattering intensity $I(q)$ was recorded as a function of the scattering vector q over the range 0.045 – 0.34 nm^{-1} using monochromatic X-rays with

an energy of 13.1 keV and a flux of $4 \cdot 10^{12}$ ph s^{-1} as recorded on an EigerX 4M (Dectris) detector in vacuum. Here, $q = \frac{4\pi}{\lambda} \cdot \sin(\theta)$, with λ the X-ray wavelength (0.95 nm) and 2θ the angle in degrees between the incident beam and the detected scattered wave. Samples were measured in a quartz glass capillary with a diameter of 1.5 mm at 20 °C. Samples were measured for 21 frames of 1 second each under flow to minimize radiation damage. Standard corrections were applied automatically at Diamond (empty capillary and solvent contributions, absolute scale corrections, *etc*.).

Fluorescence microscopy

TIRF images were acquired with a Nikon N-STORM microscopy system. Fluorescence was collected using a Nikon×100, 1.4NA oil immersion objective and passed through a quad-band pass dichroic filter (97335 Nikon). Cy3 was excited using a 561 nm laser while Cy5 was excited using a 647 nm laser. Images were recorded with an EMCCD camera (ixon3, Andor, pixel size 0.17 μm). The samples were prepared from a 0.25 mM stock by diluting with 2 mM NaOH solution to the desired concentration. Samples were imaged in a μ-Slide 8 Well plate with No. 1.5 glass coverslip bottom suitable for microscopy.

Cryo electron microscopy sample preparation

Cryo preparations of nBTA samples were accomplished with a Vitrobot Mark IV (FEI Eindhoven, The Netherlands) by following our standard protocol for aqueous surfactant solutions. This involves application of 4 µL of the aqueous sample solution to glow-discharged (60 sec, 10 mA) Quantifoil R1/4 holey carbon grids at 22 °C and 100% humidity, followed by automated blotting and plunging into liquid ethane at its freezing point.

Cryo-TEM data acquisition and analysis

Images were acquired on a FEI Talos Arctica transmission electron microscope operated at 200 kV equipped with a Falcon3EC direct electron detector operated in linear mode. Manual data acquisition was conducted using low-dose conditions with TIA software (FEI Eindhoven, Netherlands) at a nominal magnification of 28,000x, corresponding to a pixel size of 3.75 Å.

Automated data acquisition for subsequent single particle analysis was conducted at a nominal magnification of 57,000x, corresponding to a calibrated pixel size of 1.79 Å, with EPU (v2.12, FEI Eindhoven, Netherlands). A total fluence of 40 el/Å^2 was accumulated during an exposure of 1.52 s, fractionated into movies of 10 frames.

Data analysis was conducted with cryoSPARC (version 4.4.0).[5] Movies were initially aligned by Patch Motion Correction followed by Patch CTF estimation. Manually selected segments of filamentous polymers were subjected to reference-free 2D classification to generate templates for reference-based filament picking. A filament diameter of 60 Å was estimated from the images, filaments were traced with a separation distance of 120 Å. Segments were extracted with a box size of 240 px, fourier-cropped to 120 px giving a pixel size of 3.58 Å. Iterative cycles of reference-free 2D classification were conducted to select the final set of particle images with a circular mask diameter of 300 Å.

Hydrogen Deuterium Exchange Mass Spectrometry (HDX-MS)

Hydrogen deuterium exchange mass spectrometry (HDX-MS) experiments were carried out using a Xevo™ G2 QTof mass spectrometer (Waters) with a capillary voltage of 2.7 kV, a sampling cone voltage of 20 V and extraction cone voltage of 4.0 V. The source temperature was set at 100 °C, the desolvation temperature at 400 °C, the cone gas flow at 10 L h^{-1} and the desolvation gas flow at 100 L h^{-1}. The sample solutions subjected to HDX were introduced into the mass spectrometer using a Harvard syringe pump (11 Plus, Harvard Apparatus) at a flow rate of 50 µL min^{-1}. Samples of **nBTA** and surfactants were prepared at 0.5 mM of **nBTA** and diluted 100 times with D_2O (including 0.5 mM sodium acetate to facilitate detection), resulting in a final concentration of 5 µM. The samples were stored at room temperature during the experiment. The H/D exchange was followed over time and the percentages of the different deuterated isotopes were calculated. The distribution of doubly charged ions ($[BTA+2Na]^{2+}$) were used for the calculations, and the theoretical isotopic distributions were calculated with an IsoPro Software.

All **nBTA** species observed have isotopes that overlap with more deuterated analogues. For example, **nBTA** 3D has its first isotope with the same mass as monoisotopic mass of **nBTA** 4D, its second isotope with the same mass as the monoisotopic mass of **nBTA** 5D and its third isotope with the same mass as the monoisotopic mass of **nBTA** 6D.

Table S1: Theoretical isotope distribution of the doubly charged nBTA as calculated with the IsoPro Software, including a graphical representation. This would be the isotope distribution without the presence of D_2O.

$[BTA+2Na]^{2+}$	
m/z	Relative abundance
666.96	1
667.46	0.80
667.96	0.36
668.47	0.11
668.97	0.03
669.47	0.01

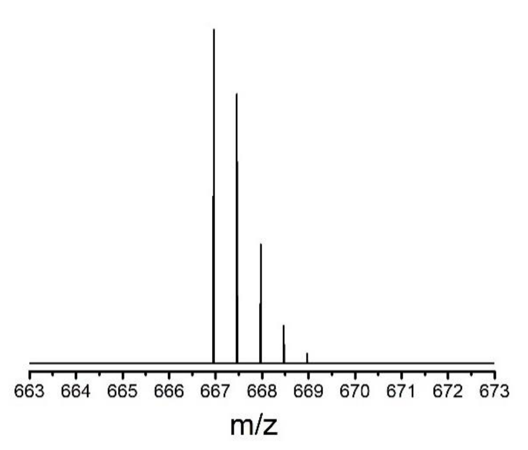

Although **nBTA** 3D and 6D are the most abundant species in the case of **nBTA** in D_2O, the isotopes of 1D, 2D, 4D and 5D were also taken into account for the calculation. With the distribution values given in Table S1, we could calculate the intensities of the isotopes and subtract these from the measured intensities at each m/z value. For example, to get the intensity of **nBTA** 3D without the influence of isotopes, we would have to subtract the first isotope of **nBTA** 2D and the second isotope of **nBTA** 1D from the measured intensity at the monoisotopic m/z of **nBTA** 3D. We assume that the deuterated **nBTA**s have the same isotope distribution as the non-deuterated **nBTA**. The corrected intensity (I_{nBTAnD_c}) of the monoisotopic peaks without the influence of the isotopes can be calculated with the constants in the table above and the following set of equations:

$$I_{nBTA1D\_c} = I_{667.46}$$

$$I_{nBTA2D\_c} = I_{667.96} - 0.80 I_{nBTA1D\_c}$$

$$I_{nBTA3D\_c} = I_{668.47} - 0.80 I_{nBTA2D\_c} - 0.36 I_{nBTA1D\_c}$$

$$I_{nBTA4D\_c} = I_{668.97} - 0.80 I_{nBTA3D\_c} - 0.36 I_{nBTA2D\_c} - 0.11 I_{nBTA1D\_c}$$

$$I_{nBTA5D\_c} = I_{669.47} - 0.80 I_{nBTA4D\_c} - 0.36 I_{nBTA3D\_c} - 0.11 I_{nBTA2D\_c} - 0.03 I_{nBTA1D\_c}$$

$$I_{nBTA6D\_c} = I_{669.97} - 0.80 I_{nBTA5D\_c} - 0.36 I_{nBTA4D\_c} - 0.11 I_{nBTA3D\_c} - 0.03 I_{nBTA2D\_c} - 0.01 I_{nBTA1D\_c}$$

With $I_{667.46}$, $I_{667.96}$, $I_{668.47}$, $I_{668.97}$, $I_{669.47}$ and $I_{669.97}$ the intensity at m/z = 667.46, 667.96, 668.47, 668.97, 669.47 and 669.97, respectively.

Surface to volume ratios

For the comparison of the spectroscopic results (UV-vis and CD) with the microscopic data we estimate by some fundamental calculations the surface to volume ratio of both measurement techniques. In the case of a 1 mm quartz cuvette, we consider all sides (water – air and water – quartz) as hydrophilic to hydrophobic interfaces. Therefore, the surface can be calculated as in good estimation as a cuboid. The values were defined by the Hellma cuvette or calculated from the volume (300 µL). In the case of the cryo-TEM grid, the hole size is defined by the Quantifoil R1/4 holey carbon grids (1.4 µm diameter). An average ice thickness of 100-200 nm was estimated. The ice volume was calculated based on a cylindric shape, in which only the top and bottom sides were considered as air-water interfaces.

Cuvette (1 x 9.5 x $Height_{300\ \mu L}$ mm):

$$\mathrm{Height}_{300\ \mu\mathrm{L}} = \frac{\mathrm{V}}{\mathrm{A_g}} = \frac{300\ \mathrm{mm}^3}{1\ \mathrm{mm} * 9.5\ \mathrm{mm}} = 31.6\ \mathrm{mm}^2$$

$$\begin{aligned}\mathrm{Surface} = \mathrm{S} &= 2(1\ \mathrm{mm} * 9.5\ \mathrm{mm}) + 2(9.5\ \mathrm{mm} * 31.6\ \mathrm{mm}) + 2(1\ \mathrm{mm} * 31.6\ \mathrm{mm}) \\ &= 682\ \mathrm{mm}^2\end{aligned}$$

$$\mathrm{Surface\ to\ Volume} = \frac{\mathrm{S}}{\mathrm{V}} = \frac{682\ \mathrm{mm}^2}{300\ \mathrm{mm}^3} = \mathbf{2.3}\ \frac{1}{\mathrm{mm}}$$

Cryo-TEM grid hole (1.4 µm diameter, 100-200 nm ice thickness)

$$Area = A = \pi r^2 = \pi(0.7\ \mu m)^2 = \frac{49}{100}\pi\ \mu m^2$$

$$Surface = S = 2 * A = \frac{98}{100}\pi\ \mu m^2$$

$$Volume_{100\ nm} = A * h = \frac{49}{100}\pi\ \mu m^2 * 0.1\ \mu m = \frac{49}{1000}\pi\ \mu m^2$$

$$Volume_{200\ nm} = A * h = \frac{49}{100}\pi\ \mu m^2 * 0.2\ \mu m = \frac{49}{500}\pi\ \mu m^2$$

$$Surface\ to\ Volume_{100\ nm} = \frac{S}{V_{100\ nm}} = \frac{\frac{98}{100}\pi\ \mu m^2}{\frac{49}{1000}\pi\ \mu m^2} = \mathbf{20}\ \frac{1}{\mu m}$$

$$Surface\ to\ Volume_{200\ nm} = \frac{S}{V_{200\ nm}} = \frac{\frac{98}{100}\pi\ \mu m^2}{\frac{49}{500}\pi\ \mu m^2} = \mathbf{10}\ \frac{1}{\mu m}$$

The surface to volume ratio of the cryo-TEM grid is **5-10** times higher compared to the surface to volume ratio of the quartz cuvette. This can indicate experimental differences between both techniques.

2. Synthesis

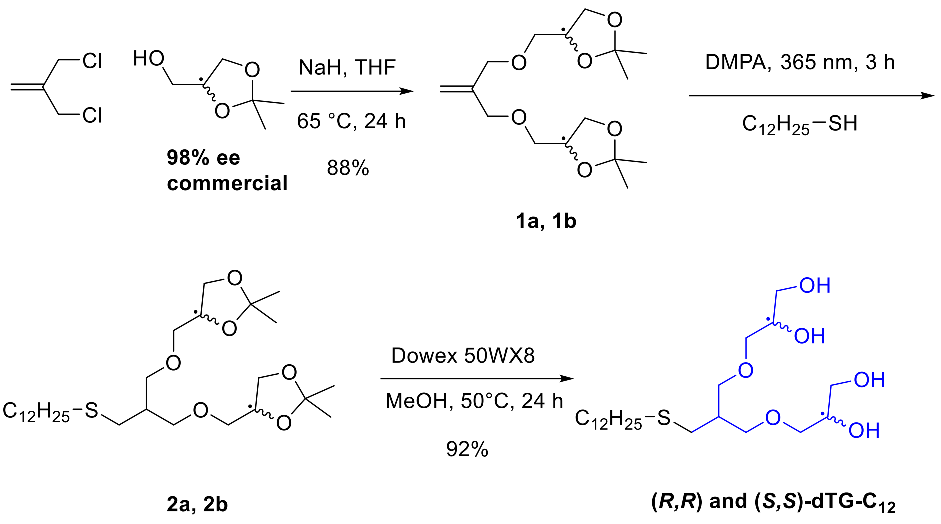

Scheme S1: Synthetic procedure for (*R,R; S,S*) **dTG-C**$_{12}$.

1 (a, b)

Sodium hydride (5.0 eq) was dissolved under Argon conditions and ice cooling in THF (50 mL). Solketal (2.1 eq, *R/S*), dissolved in THF (30 mL) and catalytic amounts of 15-Crown-5, potassium iodide and 18-crown-6 were added to the solution. 1,1-Bis(chloromethyl)ethylene (1.0 eq) was added slowly to the reaction mixture. The reaction was carried out at 65 °C overnight. The progress of the reaction was monitored by thin layer chromatography (10% EtOAc in hexane). After the reaction was completed, the THF was removed under reduced pressure and the crude was three times extracted using water/DCM (3 x 100 mL). The combined organic layers were dried over Na_2SO_4 and concentrated. The product was purified by column chromatography using hexane/EtOAc (0% → 10%).

(1a): NaH (5.0 eq, 3.80 g, 90.0 mmol), Solketal (*R*, 98% ee, 2.1 eq, 5.03 g, 37.8 mmol), Bis(chloromethyl)ethylene (1.0 eq, 2.25 g, 18.0 mmol). 90%.

^{1}H NMR (500 MHz, MeOD) δ 5.20 – 5.19 (m, 2H), 4.28 – 4.23 (m, 2H), 4.11 – 4.02 (m, 6H), 3.73 (dd, *J* = 8.3, 6.4 Hz, 2H), 3.54 – 3.44 (m, 4H), 1.38 (s, 6H), 1.33 (s, 6H) ppm.

^{13}C NMR (151 MHz, MeOD) δ 114.69, 110.39, 76.03, 72.78, 72.01, 67.40, 26.89, 25.51 ppm.

MS (ESI) m/z = 339.1793 $[M+Na]^+$ (calcd. for $C_{16}H_{28}NaO_6$: 339.1886).

(1b): NaH (5.0 eq, 3.83 g, 90.0 mmol), Solketal (*S*, 98% ee, 2.1 eq, 5.07 g, 37.8 mmol), Bis(chloromethyl)ethylene (1.0 eq, 2.21 g, 18.0 mmol). 88%.

^{1}**H NMR** (500 MHz, MeOD) δ 5.20 – 5.19 (m, 2H), 4.28 – 4.23 (m, 2H), 4.11 – 4.01 (m, 6H), 3.73 (dd, *J* = 8.3, 6.4 Hz, 2H), 3.54 – 3.42 (m, 4H), 1.38 (s, 6H), 1.33 (s, 6H) ppm.

^{13}**C NMR** (151 MHz, MeOD) δ 144.11, 114.83, 110.53, 76.17, 72.92, 72.16, 67.55, 27.03, 25.65 ppm.

MS (ESI) m/z = 339.1824 $[M+Na]^+$ (calcd. for $C_{16}H_{28}NaO_6$: 339.1886).

2 (a, b)

1a/b (1.0 eq) was dissolved in MeOH (40 mL). Argon was bubbled through the solution for 10 min. 1-Dodecanethiol (1.2 eq) and DMPA (0.15 eq) were added to the reaction mixture. The reaction was carried out with UV light (365 nm) for 3 hours. The progress of the reaction was monitored by thin layer chromatography. After the reaction was completed, the MeOH was removed under reduced pressure and the crude was three times extracted using water/DCM. The combined organic layers were dried over Na_2SO_4 and concentrated. The crude product was not purified any further and was directly used in the following reaction step.

(2a): 1a (1.0 eq, 4.0 g, 12.6 mmol), 1-Dodecanethiol (1.2 eq, 3.06 g, 15.1 mmol) and DMPA (0.15 eq, 0.49 g, 1.89 mmol).

(2b): 1b (1.0 eq, 2.9 g, 9.20 mmol), 1-Dodecanethiol (1.2 eq, 2.23 g, 11.0 mmol) and DMPA (0.15 eq, 0.17 g, 1.38 mmol).

(*R*,*R*)- and (*S*,*S*)-dTG-C$_{12}$

2a/b (1.0 eq) was dissolved in MeOH (40 mL). Dowex 50WX8 (50 w%) and a catalytic amount of water were added to the solution. The reaction was carried out at 50 °C overnight. The progress of the reaction was monitored by thin layer chromatography using 10% methanol in DCM. After the reaction was completed, the reaction mixture was filtrated and the MeOH was removed under reduced pressure. The product was purified by column chromatography using DCM/MeOH (0% → 10%).

((*R*,*R*)-dTG-C$_{12}$): 2b (1.0 eq, 3.60 g, 6.95 mmol), Dowex 50WX8 (50 w%, 1.80 g). 91%.

^{1}H NMR (600 MHz, MeOD) δ 3.77 – 3.74 (m, 2H), 3.60 – 3.45 (m, 10H), 3.44 – 3.42 (m, 2H), 2.60 (d, *J* = 6.7 Hz, 2H), 2.51 (t, *J* = 7.3 Hz, 2H), 2.06 (p, *J* = 6.1 Hz, 1H), 1.58 (p, *J* = 7.3 Hz, 2H), 1.42 – 1.37 (m, 2H), 1.36 – 1.25 (m, 16H), 0.90 (t, *J* = 7.0 Hz, 3H) ppm.

^{13}C NMR (151 MHz, MeOD) δ 73.56, 72.21, 72.06, 64.56, 41.22, 33.63, 33.08, 32.00, 30.77, 30.48, 30.38, 29.90, 23.74, 14.46 ppm.

MS (ESI) m/z = 461.2908 $[M+Na]^+$ (calcd. for $C_{22}H_{46}NaO_6S$: 461.3015)

((*S*,*S*)-dTG-C$_{12}$): 2a (1.0 eq, 3.90 g, 7.53 mmol), Dowex 50WX8 (50 w%, 1.95 g). 90%.

^{1}H NMR (600 MHz, MeOD) δ 3.77 – 3.74 (m, 2H), 3.60 – 3.47 (m, 10H), 3.44 – 3.42 (m, 2H), 2.60 (d, *J* = 6.7 Hz, 2H), 2.51 (t, *J* = 7.3 Hz, 2H), 2.06 (hept, *J* = 6.0 Hz, 1H), 1.58 (p, *J* = 7.3 Hz, 2H), 1.40 (t, *J* = 7.5 Hz, 2H), 1.30 (d, *J* = 3.7 Hz, 16H), 0.90 (t, *J* = 7.0 Hz, 3H) ppm.

^{13}C NMR (151 MHz, MeOD) δ 72.24, 70.88, 70.74, 63.24, 39.90, 32.30, 31.76, 30.68, 29.46, 29.40, 29.16, 29.05, 28.58, 22.42, 13.13 ppm.

MS (ESI) m/z = 461.2913 $[M+Na]^+$ (calcd. for $C_{22}H_{46}NaO_6S$: 461.3015)

Cy3-dTG-C12

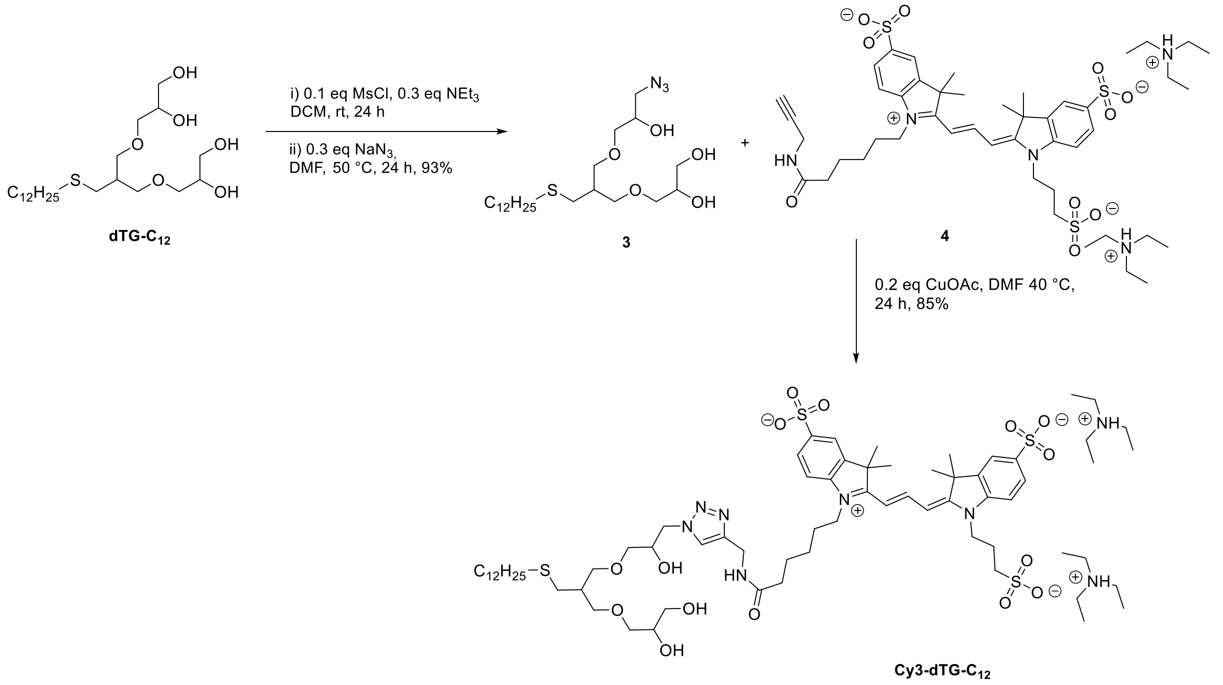

Scheme S2: Synthetic procedure for Cy3-dTG-C12.

(3)

dTG-C12 (10 eq, 200 mg, 0.50 mmol) was dissolved in DCM (20 mL). Under ice cooling NEt_3 (3.0 eq, 20.8 µL, 0.15 mmol) was added slowly to the reaction mixture. After 30 min of stirring MsCl (1.0 eq, 4.00 µL, 0.05 mmol) was added slowly the reaction mixture. The reaction was carried out over night at room temperature. The progress of the reaction was monitored using thin layer chromatography (10% methanol in DCM). After the removal of DCM, the residue was dissolved in DMF (20 mL) and NaN_3 (3.0 eq, 8.90 mg, 0.10 mmol) was added and the reaction was heated up to 50 °C. After 24 h, the reaction was completed, and the solvent was removed. The residue was purified by column chromatography, using DCM/MeOH (0% → 10%). **3** was obtained with a yield of 93%.

^{1}H NMR (700 MHz, MeOD) δ 3.88 – 3.85 (m, 1H), 3.75 (p, *J* = 5.4 Hz, 1H), 3.65 – 3.28(m, 12H), 2.60 – 2.50 (m, 4H), 2.07 – 2.05 (m, 1H), 1.58 (p, *J* = 7.4 Hz, 2H), 1.41 – 1.30 (m, 18H), 0.90 (t, *J* = 7.0 Hz, 3H) ppm.

^{13}C NMR (176 MHz, MeOD) δ 73.58, 72.46, 72.22, 72.10, 69.37, 64.57, 62.36, 41.18, 37.24, 37.17, 33.62, 33.55, 33.08, 33.00, 31.96, 31.89, 30.78, 30.71, 30.48, 30.37, 29.90, 29.81, 23.74, 23.66, 14.46, 14.38 ppm.

MS (ESI) m/z = 486.3074 $[M+Na]^+$ (calcd. for $C_{22}H_{45}N_3NaO_5S$: 486.3080).

Cy3-dTG-C12

3 (1.0 eq, 1.80 mg, 3.90 µmol) and Cy3-alkyne 4 (0.7 eq, 2.32 mg, 2.71 µmol) was dissolved in DMF (5 mL). Cu(I)Ac (0.2 eq, 1.01 mg, 0.80 µmol) was added to the reaction mixture. The reaction was carried out over night at 40 °C and monitored by thin layer chromatography. After the completion of the reaction, the solvent was removed under reduced pressure and the residue was dissolved in water and dialyzed (100-500 D) against water for 2 days (changing the water thrice a day). The residue was lyophilized, and the product **Cy3-dTG-C12** was obtained in a yield of 85% with a degree of functionalization of 40%.

^{1}H NMR (700 MHz, MeOD) δ 8.62 – 8.58 (m, 1H), 7.98 – 7.94 (m, 5H), 7.53 – 7.342 (m, 2H), 6.77 – 6.72 (m, 1H), 6.64 – 6.57 (m, 1H), 4.43 – 4.41 (m, 2H), 4.23 – 4.21 (m, 3H), 3.67 – 3.45 (m, 74H), 2.63 – 2.62 (m, 3H), 2.54 – 2.52 (m, 3H), 2.30 – 2.22 (m, 4H), 1.82 (d, *J* = 6.7 Hz, 17H), 1.61 – 1.59 (m, 6H), 1.41 – 1.31 (m, 44H), 0.92 (t, *J* = 6.9 Hz, 8H) ppm.

MS (ESI) m/z = 611.2600 $[M]^{2-}$ (calcd. for $C_{57}H_{86}N_6O_{15}S_4^{2-}$: 611.2522).

3. Physicochemical Characterization of Surfactants

3.1 Critical micelle concentration of dTG-C_{12}

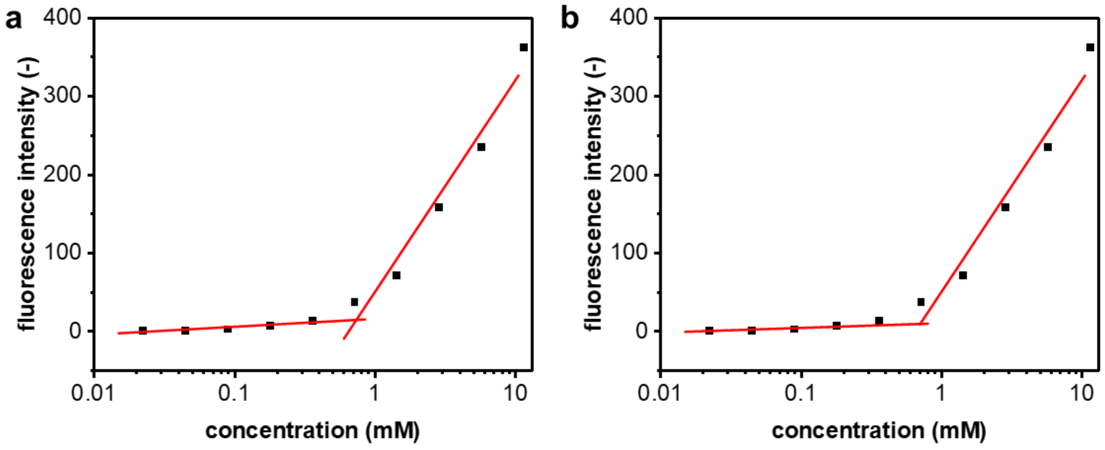

Figure S1: CMC calculation of (*S*,*S*)-/(*R*,*R*)-**dTG-C**$_{12}$ using dye-encapsulation (Nile red) followed by fluorescence spectroscopy. (a) CMC of (*S*,*S*)-**dTG-C**$_{12}$ is 0.73 mM (b) CMC of (*R*,*R*)-**dTG-C**$_{12}$ is 0.71 mM.

3.2 Spectroscopic data of dTG-C_{12} and DDM

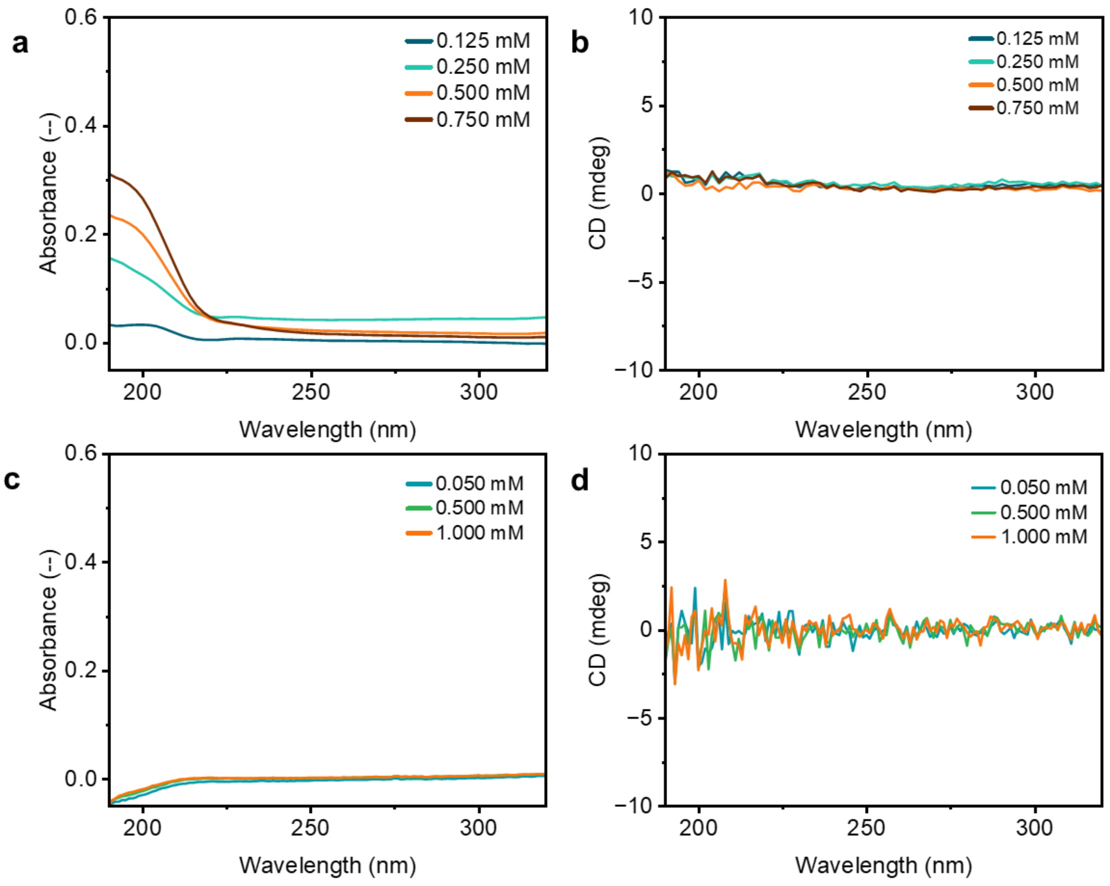

Figure S2: Spectroscopic data of **dTG-C_{12}** and **DDM** self-assembled aggregates in Milli-Q water. (*S*,*S*)-**dTG-C_{12}** in different concentrations (below and above the CMC), visualizing weak to no UV-vis absorption (a) and silent CD spectra (b) for all measured concentrations in the wavelength range of interest. **DDM** at concentrations of 0.05 mM, 0.5 mM and 1 mM, showing net zero UV-vis absorption values (c) and CD silence (d) over the wavelength range of interest.

4. Coassembly studies

4.1 Spectroscopic data of nBTA-surfactant coassemblies

4.1.1 Dilution studies

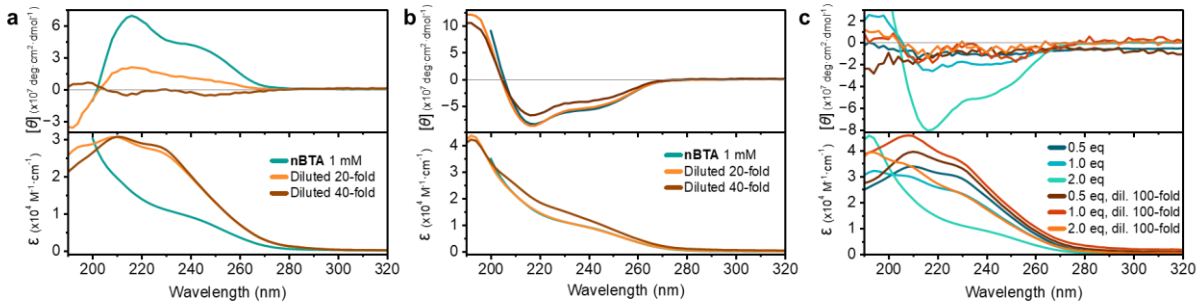

Figure S3: Molar ellipticity (top) and extinction (bottom) calculated from the CD and UV-vis absorption spectra of 1 mM **nBTA** mixed with 1.8 equivalents of (a) **DDM** and with 1.8 equivalents of (b) (*S*,*S*)-**dTG-C12**. The samples were diluted 20- and 40-fold (orange and brown lines, respectively) to test for dilution induced re-assembly of the double helical assembled structures of **nBTA**.[7] For the mixture with **DDM**, dilution of 20-fold resulted in an intermediate absorption profile and decrease in ellipticity. Further dilution of 40-fold resulted in the original UV-vis absorption profile and a slight CD intensity, resembling pristine (S)-**D-BTA** samples. In contrast, the mixture with 1.8 equivalents of (*S*,*S*)-**dTG-C12** showed consistent CD and UV-vis absorption profiles with relatively constant values for ellipticity and extinction, with slight deviations in the 40-fold diluted sample with respect to the non-diluted sample. These results indicate that the **dTG-C12** surfactant is less sensitive to dilution and retains its interaction with **nBTA**, whereas dilution of the mixtures with **DDM** indeed results in dilution-induced re-assembly of double helices due to lower surfactant-polymer interactions. We believe this difference originates from the morphologies formed by homo-assemblies of both surfactants, where **DDM** assembles into spherical micelles, whereas **dTG-C12** assembled into worm-like micelles. Hence, **dTG-C12** coordination to the polymeric assemblies of **nBTA** does not result in a high penalty of re-organization to allow for the coassembly, as homo-assembly yields a similar elongated structure. We extended the dilution study by 100-fold dilution of 0.5 mM **nBTA** samples mixed with 0.5-2.0 equivalents of (*S*,*S*)-**dTG-C12** (c). Evidently for the 2.0 equivalent sample, the 100-fold diluted sample shows an absorption profile that indicates a partial single and partial double helix composition, proving that a high enough dilution factor allows for double helix re-assembly.

4.1.2 Concentration dependent coassembly of **nBTA** with **dTG-C$_{12}$** and **DDM**

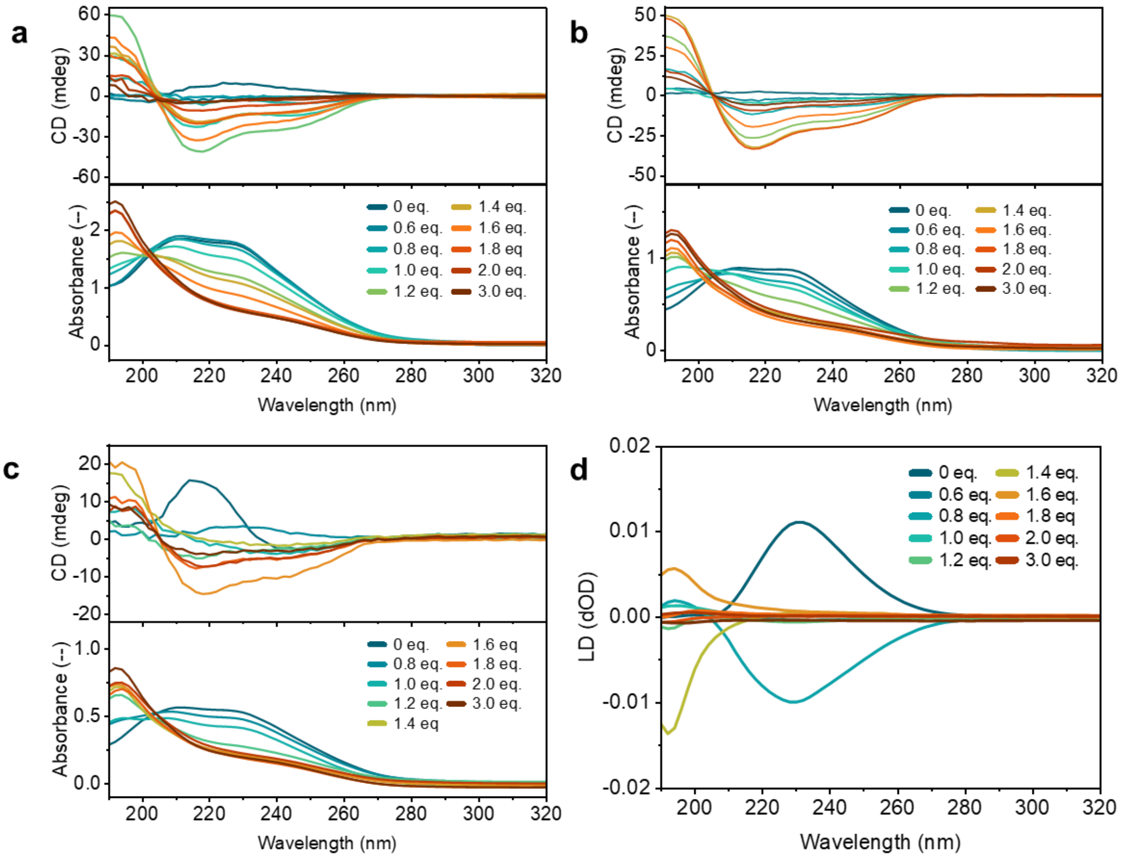

Figure S4: UV-Vis and CD spectra of **nBTA** solutions at (a) 0.05 mM, (b) 0.25 mM (c) 1 mM containing varying molar equivalents of (*S,S*)-**dTG-C$_{12}$**. The shift in the UV-vis absorption spectrum depends on both stoichiometry and concentration, as the data shows that the shift occurs at lower equivalents of surfactant for higher concentrations of **nBTA**. Some of the mixtures with low equivalents of surfactant at the higher **nBTA** concentrations show deviating CD spectra, which are attributed to linear dichroism (LD) artifacts and which is commonly observed for **nBTA** samples due to long fiber alignment in the cuvettes.[2,6] Upon mixing of surfactants, the LD artifacts diminished. The LD spectra were acquired with the same samples at **nBTA** concentrations of 1 mM.

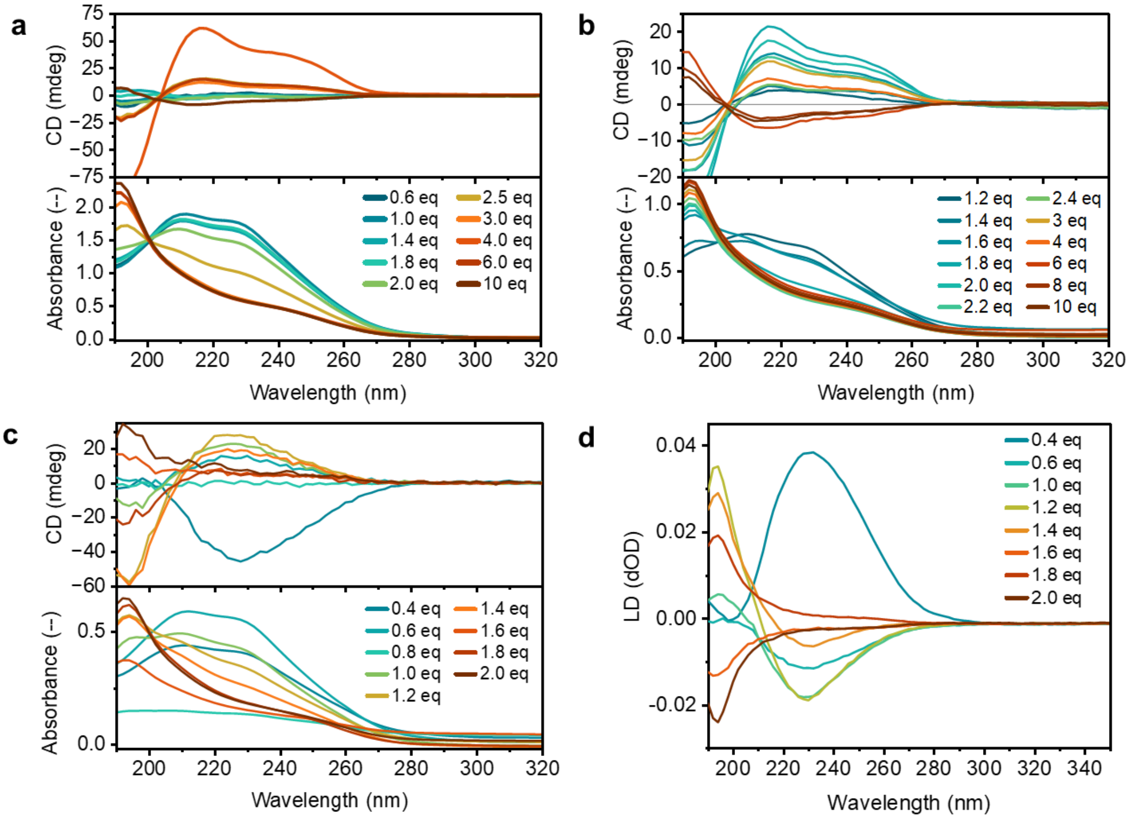

Figure S5: UV-vis and CD spectra of **nBTA** solutions at (a) 0.05 mM, (b) 0.25 mM, (c) 1 mM containing varying molar equivalents of **DDM**. In contrast to (*S*,*S*)-**dTG-C12** (Fig. S3), strong difference in the CD maximum is observed between the three concentrations, where the 0.05 mM of **nBTA** shows maximum intensity at 4.0 equivalent of **DDM** whereas the maximum is at 1.8 equivalents for the 0.25 mM of **nBTA** mixture. At 1 mM, this maximum is observed at 1.2 equivalent, but the mixtures show significant LD contributions (d) and therefore are less reliable. For the mixtures at $c_{\mathbf{nBTA}}$ = 0.05 mM and 0.25 mM, the LD contribution was similar to mixtures of **nBTA** and (*S*,*S*)-**dTG-C12**, meaning absent for mixtures of > 0.6 equivalents of surfactant.

4.1.3 (*R*,*R*)-**dTG-C12** and **nBTA** coassembly

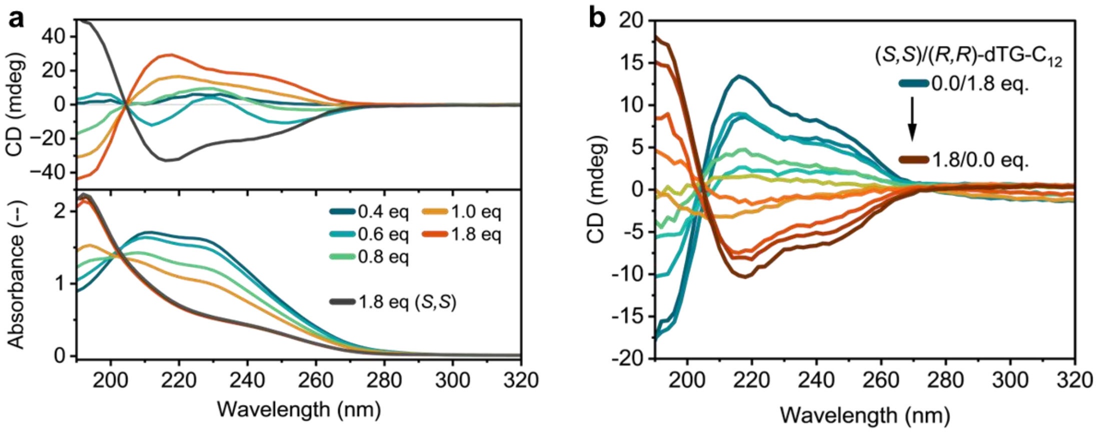

Figure S6: (a) 0.5 mM **nBTA** mixed with 0.4 – 1.8 equivalents of (*R*,*R*)-**dTG-C12** (colored lines) after two nights of equilibration. The increase in intensity up to 1.8 equivalents of the surfactant is the mirror imaged CD development with respect to that of the mixture with (S,*S*)-**dTG-C12** (black line). (b) CD spectra of mixtures of **nBTA** (0.25 mM) and various ratios of (*S*,*S*)- and (*R*,*R*)-**dTG-C12**, at a constant surfactant concentration of 0.375 mM (1.8 molar equivalents)

4.2 Relation of stereoisomers to BTA-fiber asymmetry

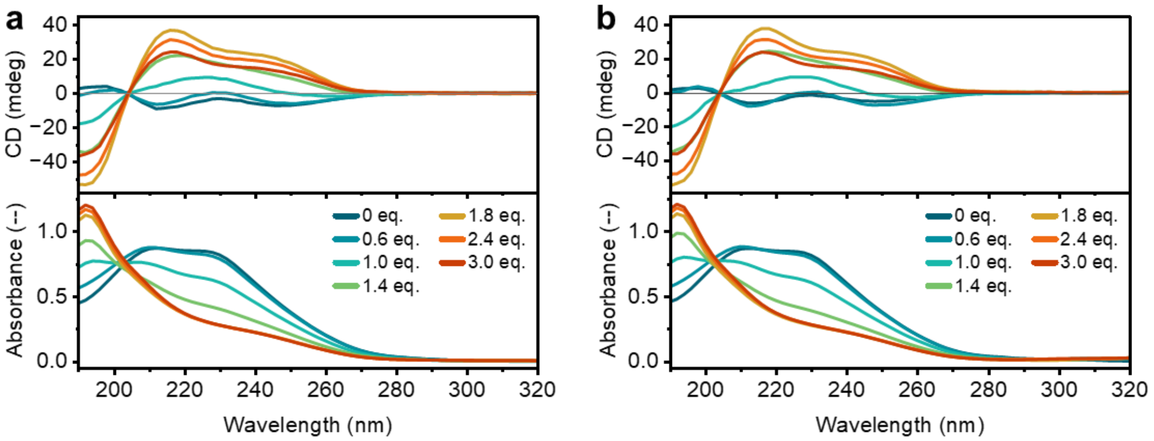

Figure S7: CD and UV-vis absorption spectra of mixtures of 0.25 mM **(*S*)-D-BTA** with various equivalents of (a) (*S*,*S*)-**dTG-C12** and (b) (*R*,*R*)-**dTG-C12**. For both enantiopure series, the course of CD and UV-vis absorption profiles are identical and always towards the positive CD sign with a maximum at 1.8 equivalents, proving that the stereocenter of **(*S*)-D-BTA** is overruling in depicting the handedness of the polymers.

4.3 Fourier-transform infrared (FTIR) spectroscopy

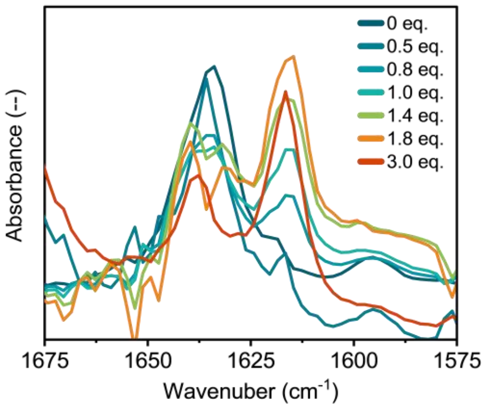

Figure S8: FTIR spectra of the amide I vibration of 1 mM of **nBTA** double helices (blue), with increasing molar equivalents of **DDM**, showing a split peak pattern with peaks positioned at 1635 cm^{-1} and 1616 cm^{-1}. The difference in the spectrum indicates a different organization of amides within the assemblies, presumably what is resulting in the double or single helical assemblies. Therefore, the FTIR data can function as one of several diagnostic methods to confirm whether a BTA assembly in water is in a double or single helical state.

4.4 Small-angle X-ray scattering (SAXS)

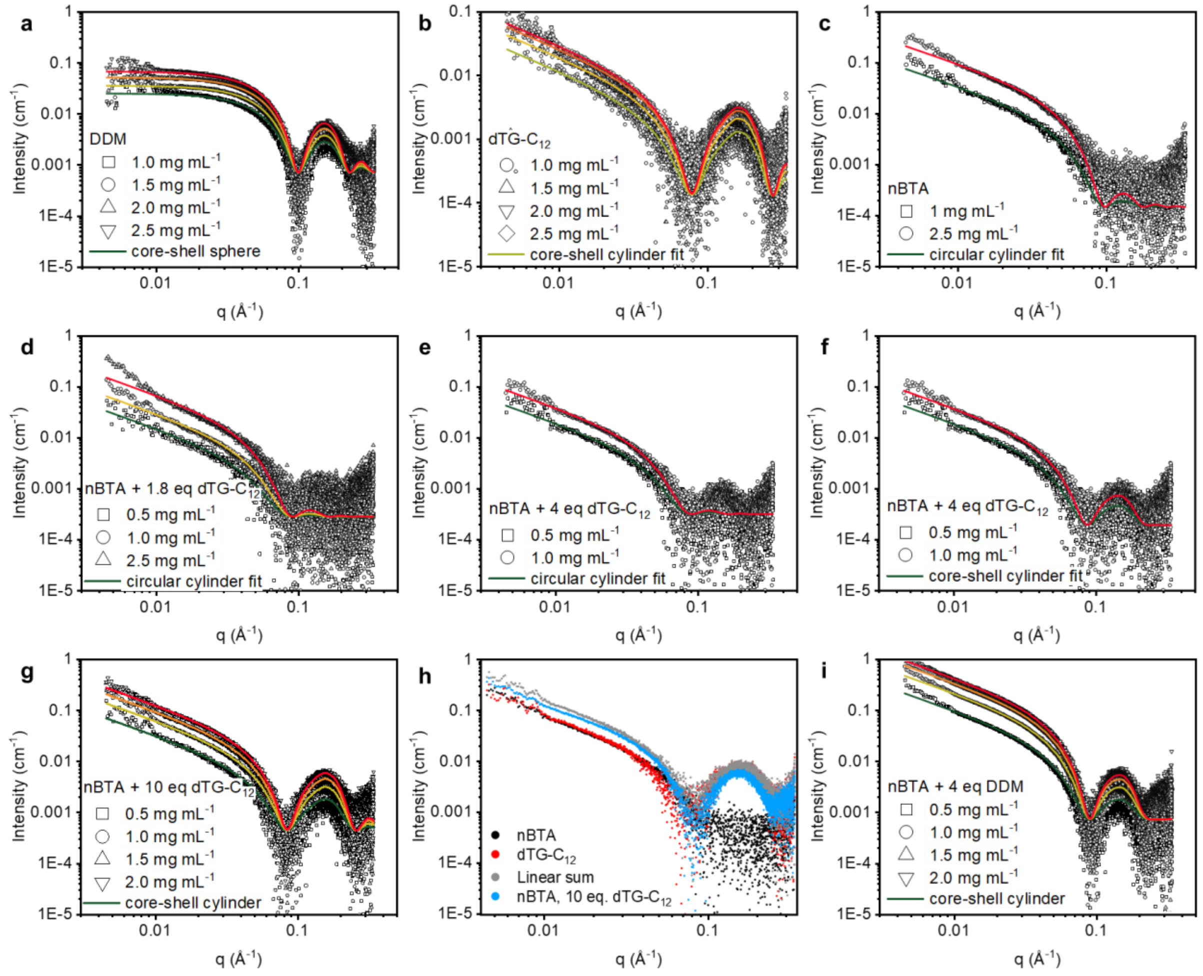

Figure S9: Collection of small-angle X-ray scattering (SAXS) profiles of (a) **DDM**, (b) **dTG-C$_{12}$** and (c) **nBTA** at multiple concentrations to exclude artifacts and confirm robustness of the assemblies over the concentration range. All scattering data was fitted to form factor models to label the type of assembly. This revealed that **DDM** assembles into spherical core-shell structures (*i.e.* spherical micelles), while **dTG-C$_{12}$** assembles into cylindrical core-shell structures – matching with the worm-like micellar morphology as observed previously and with CryoTEM. **nBTA**, as observed previously, assembles into small cross-sectional cylindrical structures. However, the high noise in the data of the higher *q* region led to an inaccurate estimation of the detailed cross-sectional structure: circular or elliptical. Various concentrations of **nBTA** were mixed with 1.8, 4.0 and 10 molar equivalents of **dTG-C$_{12}$** (ranging from 0.3 mg mL^{-1} to 8.5 mg mL^{-1} of **dTG-C$_{12}$** in solution). In all cases, the data could be fitted to cylindrical models. For the 1.8 eq. **dTG-C$_{12}$** mixture (d) data fitted best to the circular cylindrical model. For 4.0 eq. of **dTG-C$_{12}$**, the data fitted well to the circular (e) as well as core-shell cylinder fits (f), indicating that more structures in solution would adopt to the **dTG-C$_{12}$**-governed structure. For the 10 eq. **dTG-C$_{12}$** samples (g), clear core-shell cylindrical structures would be obtained at all concentrations. To judge if coassembly occurred, we took the profiles of pristine **nBTA** (1.0 mg mL^{-1}) and **dTG-C$_{12}$** (2.5 mg mL^{-1}) and calculated the linear sum of these two individual spectra. We then compared the calculated profile to the measured profile of 1.0 mg ml^{-1} **nBTA** and 10 molar equivalents of **dTG-C$_{12}$** (corresponding to 3.4 mg mL^{-1}, *i.e.* higher than the reference sample), and observed that the profiles are similar (h). Therefore, these results do not directly indicate that coassembly had occurred. Important to note here is that both pristine

nBTA and **dTG-C12** form elongated cylindrical structures, and when **nBTA** fibers would be decorated with **dTG-C12** molecules, a core-shell structure would be the result. Hence, the SAXS data for these structures is inconclusive. However, mixtures of **nBTA** with 4.0 molar equivalents of **DDM** (i) gave only elongated structures for all concentrations, while pristine **DDM** solutions only yielded spherical micelles. Hence, making the same calculation here showed that indeed new structures are formed which deviate from the calculated linear sum (see main text).

4.5 Total internal reflection fluorescence (TIRF) microscopy

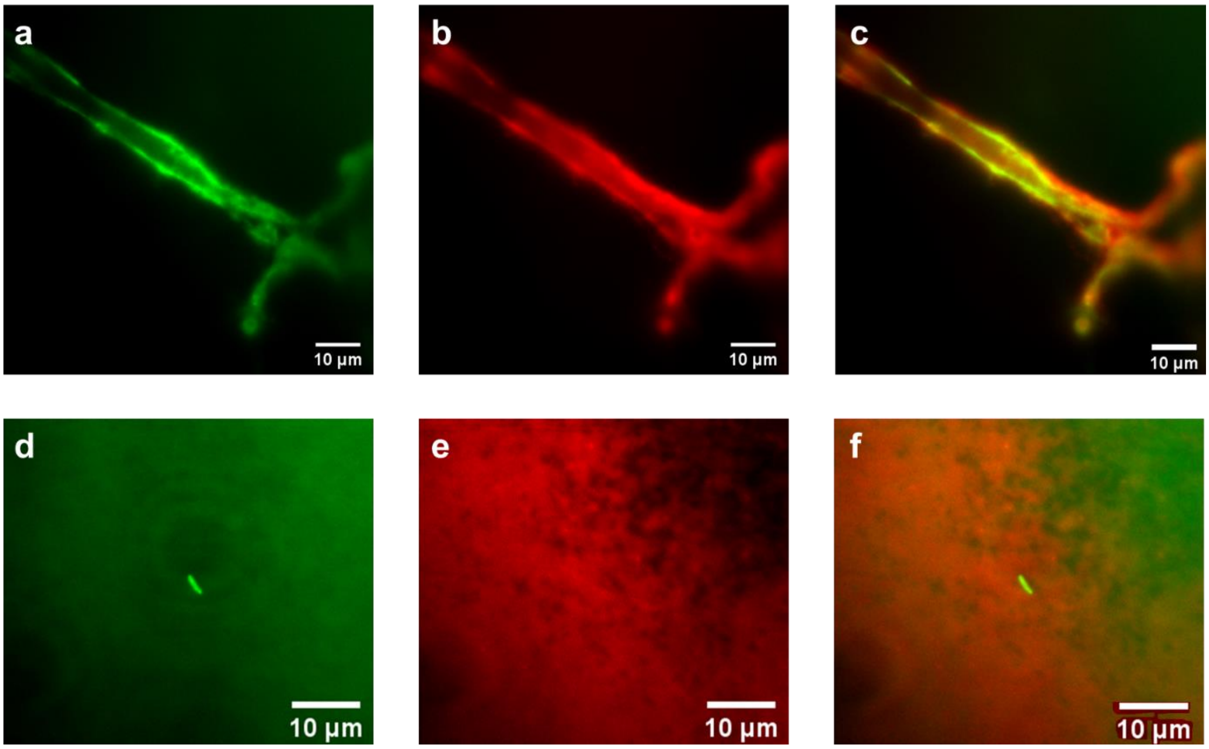

Figure S10: Total internal reflection fluorescence (TIRF) microscopy images of **nBTA** (0.05 mM) having 5 mol% of Cy5-labeled BTA,[3] mixed with 1.8 molar equivalents of **dTG-C12** having 5 mol% of the Cy3-labeled **dTG-C12**. Fibrous structures were observed both for excitation of Cy3 (a) corresponding to the **dTG-C12** structures, and Cy5 (b) corresponding to BTA structures. Overlaying the two images revealed colocalization (c). In some images, small worm-like objects were observed which only appeared upon excitation of Cy3 (d) and not for excitation of Cy5 (e), showing that some surfactant assemblies existed that were not oriented onto **nBTA**-based assemblies. Moreover, images d-f serve as a control demonstrating that assemblies containing only Cy3-labeled molecules do not show up when exciting and monitoring the Cy5.

4.6 Hydrogen-deuterium exchange – mass spectrometry (HDX-MS)

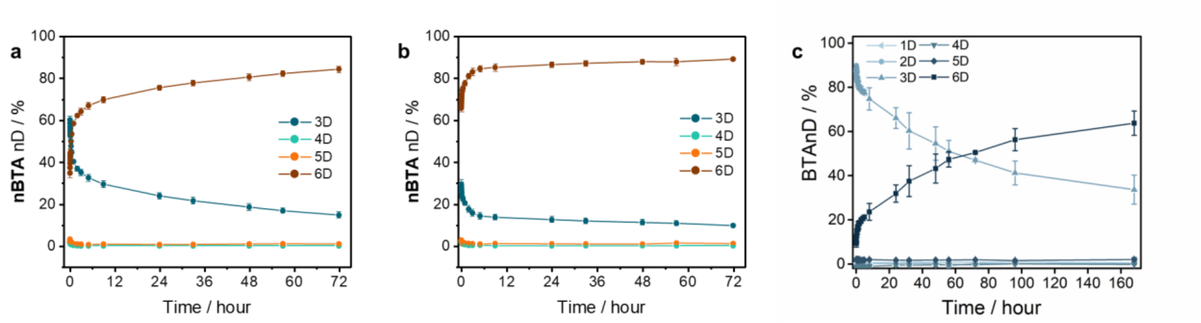

Figure S11: Hydrogen-deuterium exchange followed by mass spectrometry (HDX-MS) profiles showing all deuterated **nBTA** species acquired for mixtures of **nBTA** (0.5 mM) with 0.5 (a) and 1.0 (b) molar equivalent of (*R*,*R*)-**dTG-C**$_{12}$. (c) HDX-MS profiles for all deuterated species upon 100-fold dilution in D_2O of 0.5 mM (*S*)-**Me-BTA.** The profiles are averaged out over 3 runs, standard deviation error bars are included.

4.7 Cryo-TEM/Class sum images

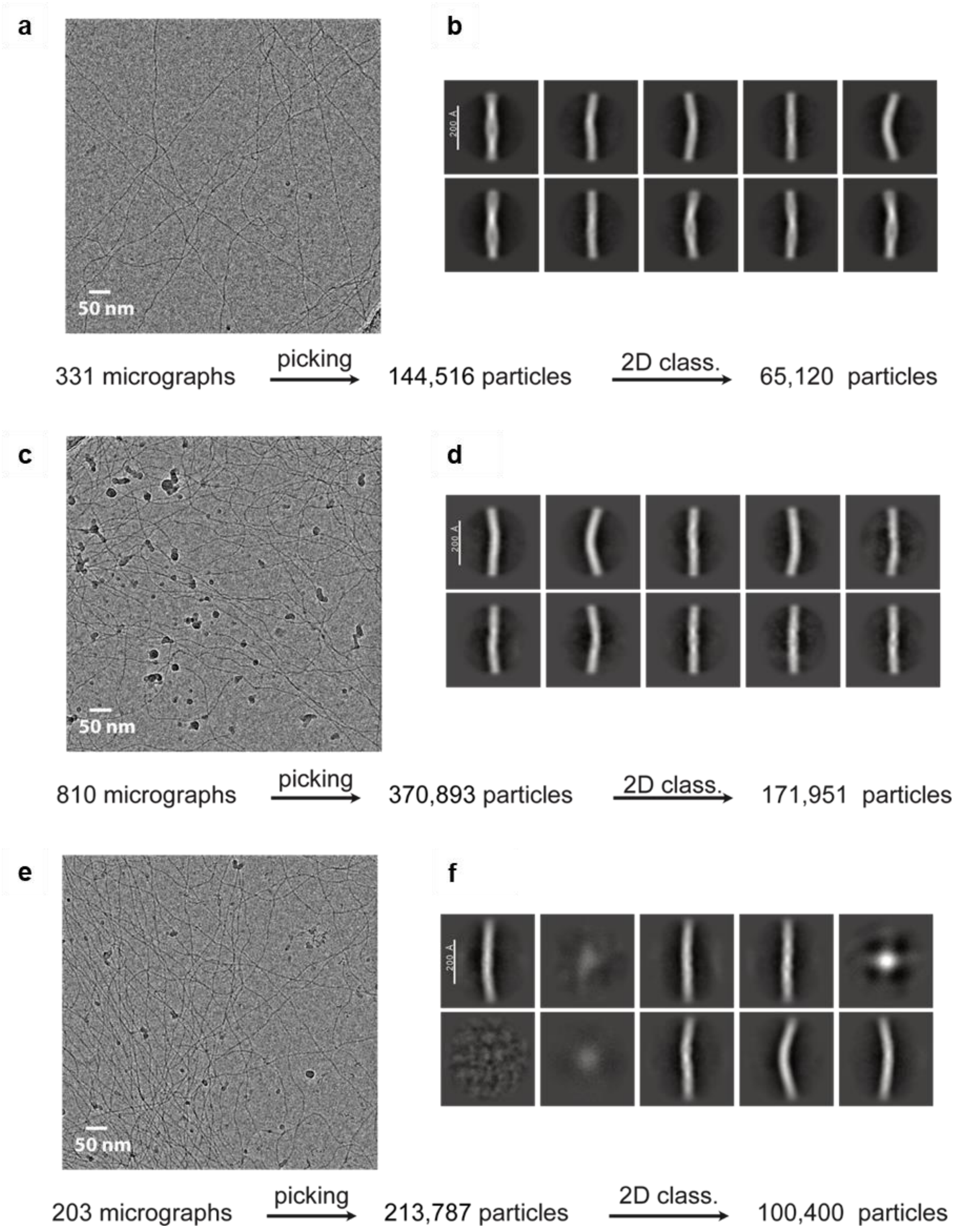

Figure S12: Data analysis workflow: After motion and CTF correction of raw movies, fibre section images (not shown) were automatically selected and cropped from micrographs (a, c, e). Fibre section images were subjected to reference-free 2D classification generating summed 2D class averages (b, d, f) which provide a strongly enhanced signal-to-noise ratio. The figure displays the results for three exemplary coassemblies of **nBTA** (0.25 mM) with 1.8 eq. **dTG-C$_{12}$** (a, b), 4.0 eq. **dTG-C$_{12}$** (c, d), and 4.0 eq. **DDM** (e, f).

The following galleries reflect the structural peculiarities of the different coassemblies. Each row shows five selected class sum images from the respective data sets, which contain the combined information of approximately 10.000 to 30.000 fibre sections.

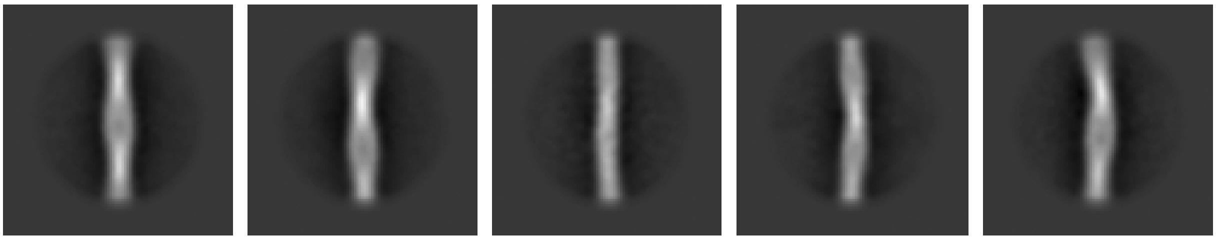

Figure S13: Image processing of cryo-TEM data of the aqueous solution of **nBTA** (0.25 mM) with 1.8 eq. of **dTG-C**$_{\mathbf{12}}$. The gallery of selected class sum images depicts the existence of two types of elongated fibers, twisted helical and straight smooth ones.

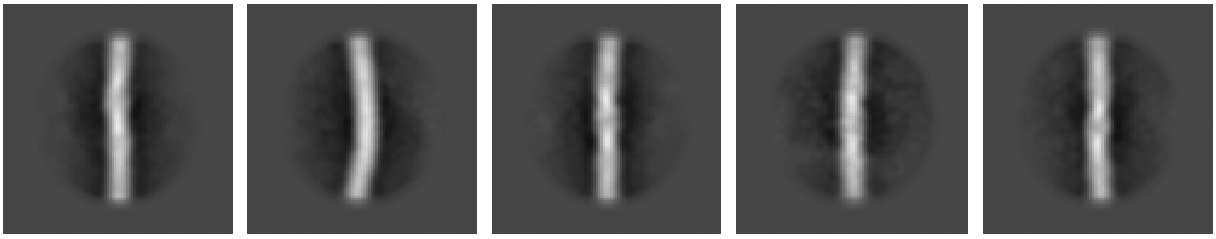

Figure S14: Image processing of cryo-TEM data of the aqueous solution of **nBTA** (0.25 mM) with 4.0 eq. of **dTG-C**$_{\mathbf{12}}$. Here, the gallery of class sum images suggests the existence of mainly smooth fibers.

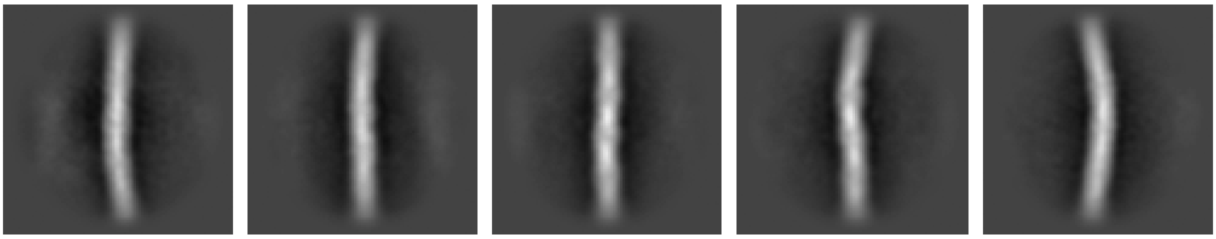

Figure S15: Image processing of cryo-TEM data of the aqueous solution of **nBTA** (0.25 mM) with 4.0 eq. of **DDM**. Again, the gallery depicts class sum images of smooth fibers.

Please note: Sometimes irregular pattern along the longitudinal axis are likely resulting from noise, which becomes more significant in the classification process when actual structural information is missing, e.g. as in the smooth fibres (Figure S14 andFigure **S15**). Such artificial density distributions are absent, when a real structure, e.g., a twisted fibre, is present (Figure **S13**).

5. References

(1) Leenders, C. M. A.; Albertazzi, L.; Mes, T.; Koenigs, M. M. E.; Palmans, A. R. A.; Meijer, E. W. Supramolecular Polymerization in Water Harnessing Both Hydrophobic Effects and Hydrogen Bond Formation. *Chem. Commun.* **2013**, *49* (19), 1963–1965.

(2) Schoenmakers, S. M. C.; Spiering, A. J. H.; Herziger, S.; Böttcher, C.; Haag, R.; Palmans, A. R. A.; Meijer, E. W. Structure and Dynamics of Supramolecular Polymers: Wait and See. *ACS Macro Lett.* **2022**, *11* (5), 711–715.

(3) Albertazzi, L.; Van Der Zwaag, D.; Leenders, C. M. A.; Fitzner, R.; Van Der Hofstad, R. W.; Meijer, E. W. Probing Exchange Pathways in One-Dimensional Aggregates with Super-Resolution Microscopy. *Science* **2014**, *344* (6183), 491–495.

(4) Cowieson, N. P.; Edwards-Gayle, C. J. C.; Inoue, K.; Khunti, N. S.; Doutch, J.; Williams, E.; Daniels, S.; Preece, G.; Krumpa, N. A.; Sutter, J. P.; Tully, A. D.; Terrill, N. J.; Rambo, R. P. Beamline B21: High-Throughput Small-Angle X-Ray Scattering at Diamond Light Source. *J. Synchrotron Radiat.* **2020**, *27*, 1438–1446.

(5) Punjani, A.; Rubinstein, J. L.; Fleet, D. J.; Brubaker, M. A. CryoSPARC: Algorithms for Rapid Unsupervised Cryo-EM Structure Determination. *Nat. Methods* **2017**, *14* (3), 290–296.

(6) Wolffs, M.; George, S. J.; Tomović, Ž.; Meskers, S. C. J.; Schenning, A. P. H. J.; Meijer, E. W. Macroscopic Origin of Circular Dichroism Effects by Alignment of Self-Assembled Fibers in Solution. *Angew. Chem., Int. Ed.* **2007**, *46* (43), 8203–8205.

(7) Su, L.; Mosquera, J.; Mabesoone, M. F. J.; Schoenmakers, S. M. C.; Muller, C.; Vleugels, M. E. J.; Dhiman, S.; Wijker, S.; Palmans, A. R. A.; Meijer, E. W. Dilution-Induced Gel-Sol-Gel-Sol Transitions by Competitive Supramolecular Pathways in Water. *Science* **2022**, *377* (6602), 213–218.

(8) Lou, X.; Schoenmakers, S. M. C.; van Dongen, J. L. J.; Garcia-Iglesias, M.; Casellas, N. M.; Fernández-Castaño Romera, M.; Sijbesma, R. P.; Meijer, E. W.; Palmans, A. R. A. Elucidating Dynamic Behavior of Synthetic Supramolecular Polymers in Water by Hydrogen/Deuterium Exchange Mass Spectrometry. *J. Polym. Sci.* **2021**, *59* (12), 1151–1161.

6. Appendix

6.1 NMR

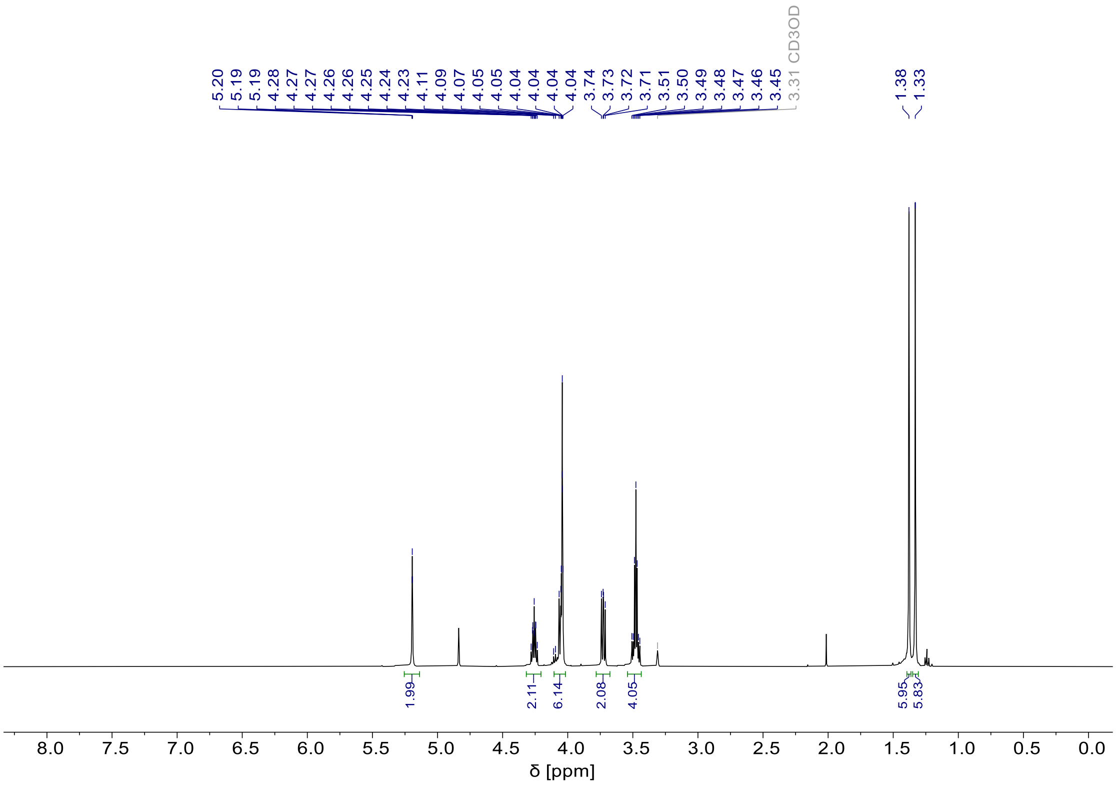

Figure S16: ^{1}H NMR (500MHz, MeOD) of **1a**.

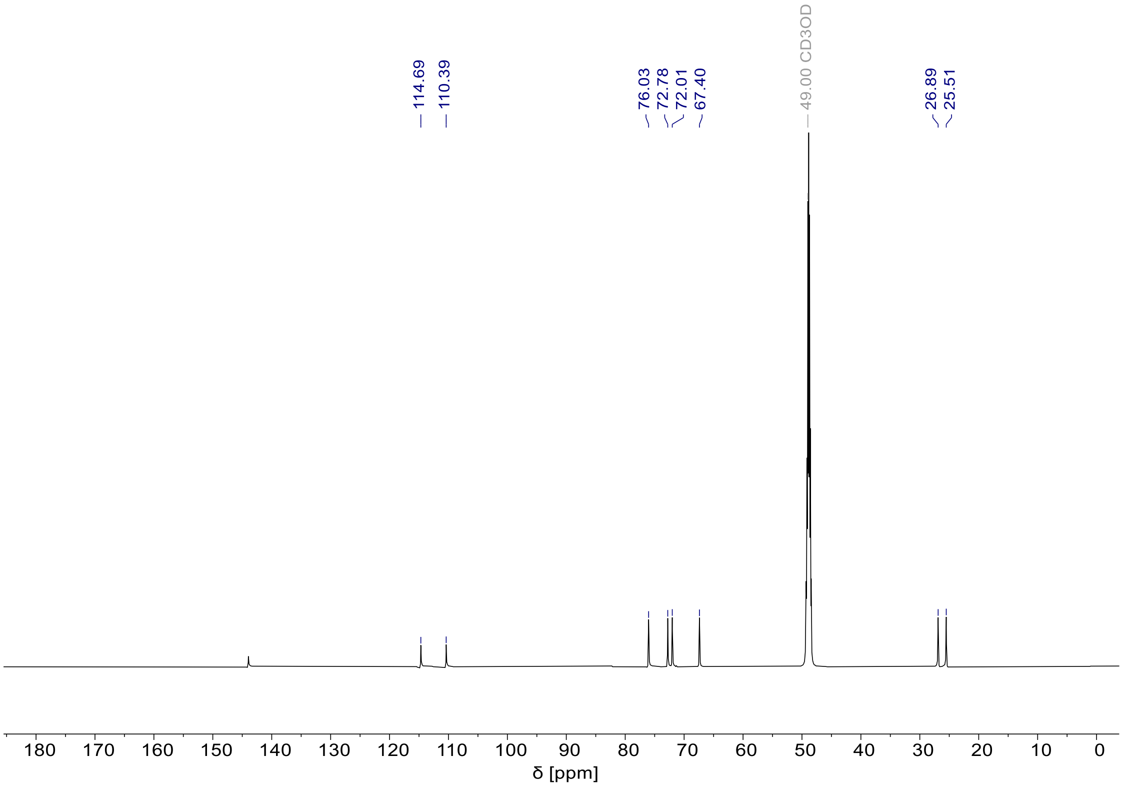

Figure S17: ^{13}C NMR (151 MHz, MeOD) of **1a**.

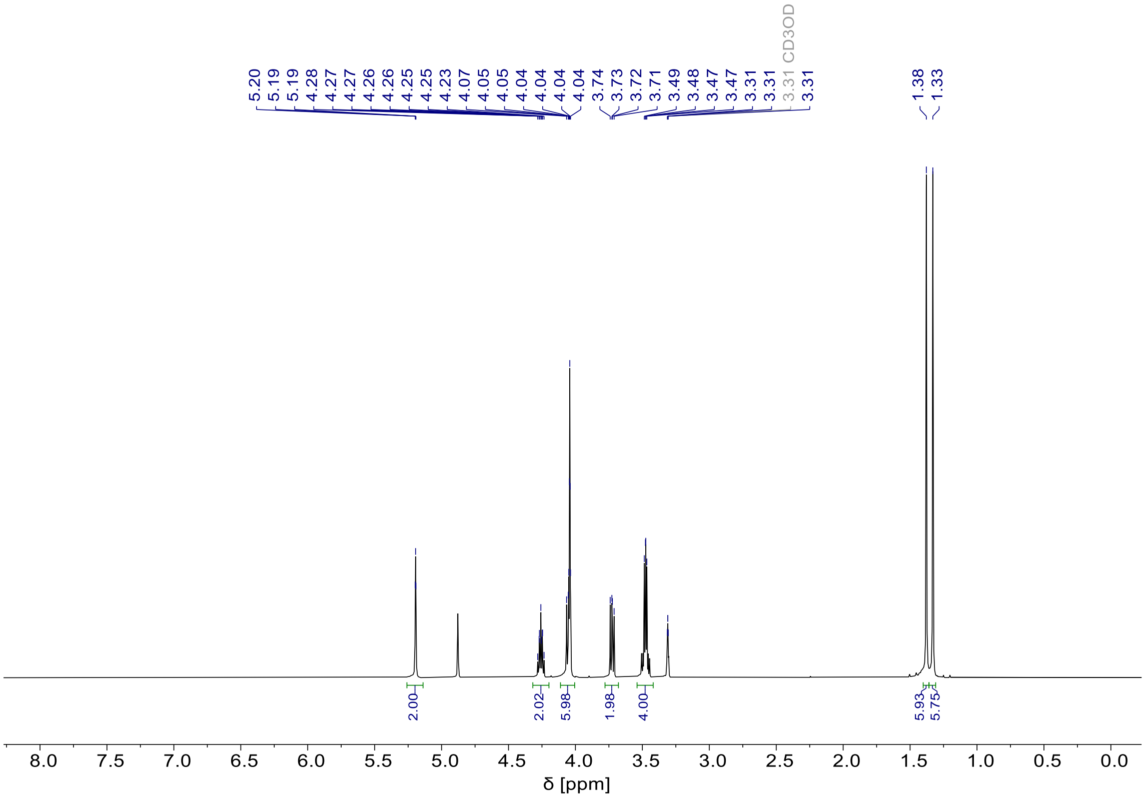

Figure S18: ^{1}H NMR (500 MHz, MeOD) of **1b**.

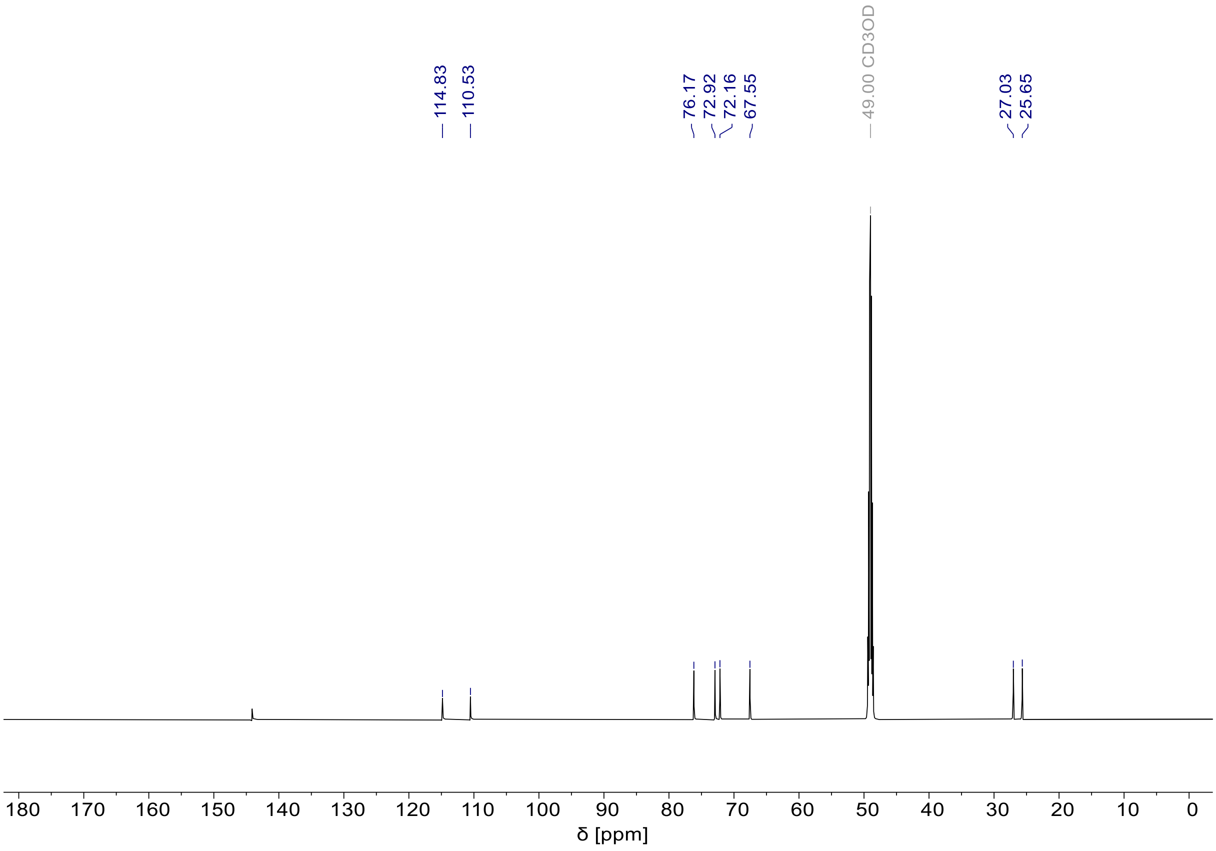

Figure S19: ^{13}C NMR (151 MHz, MeOD) of **1b**.

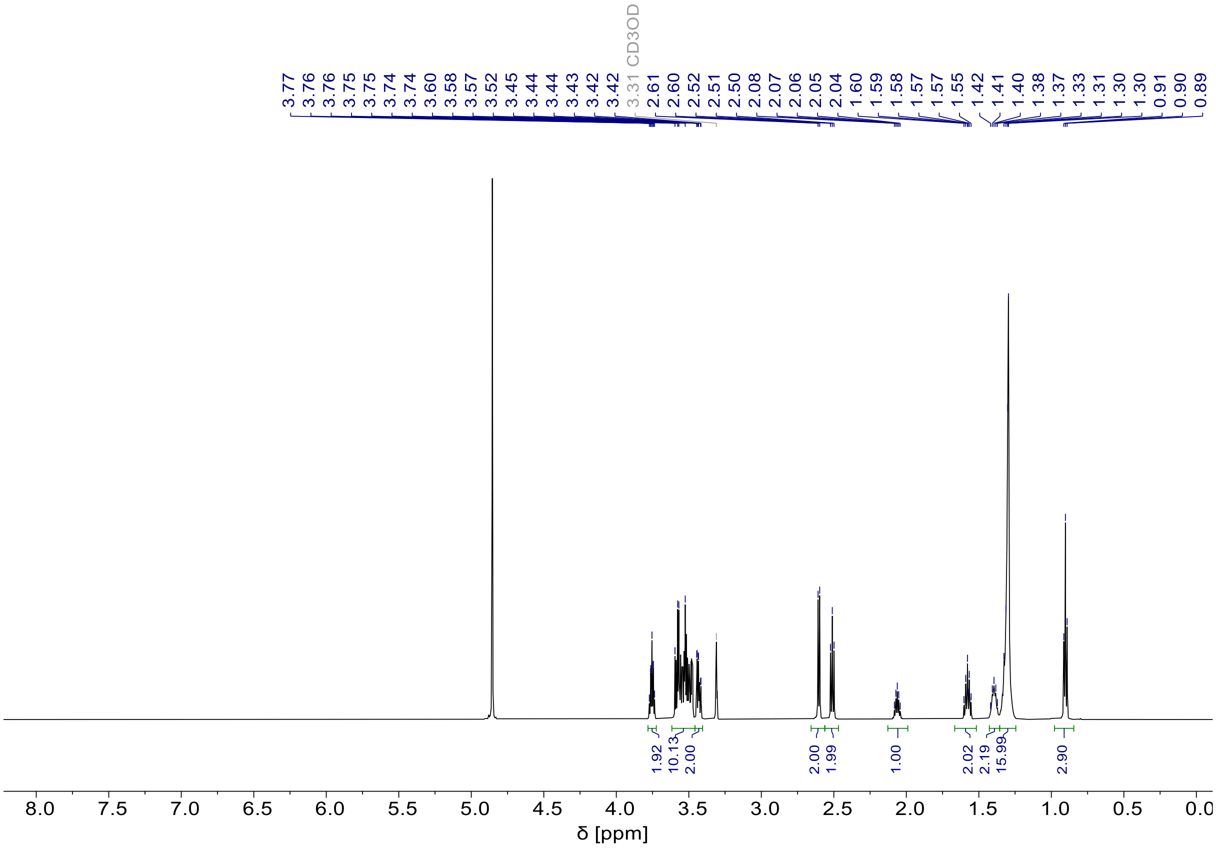

Figure S20: ^{1}H NMR (600 MHz, MeOD) of (*R*,*R*)-**dTG-C$_{12}$**.

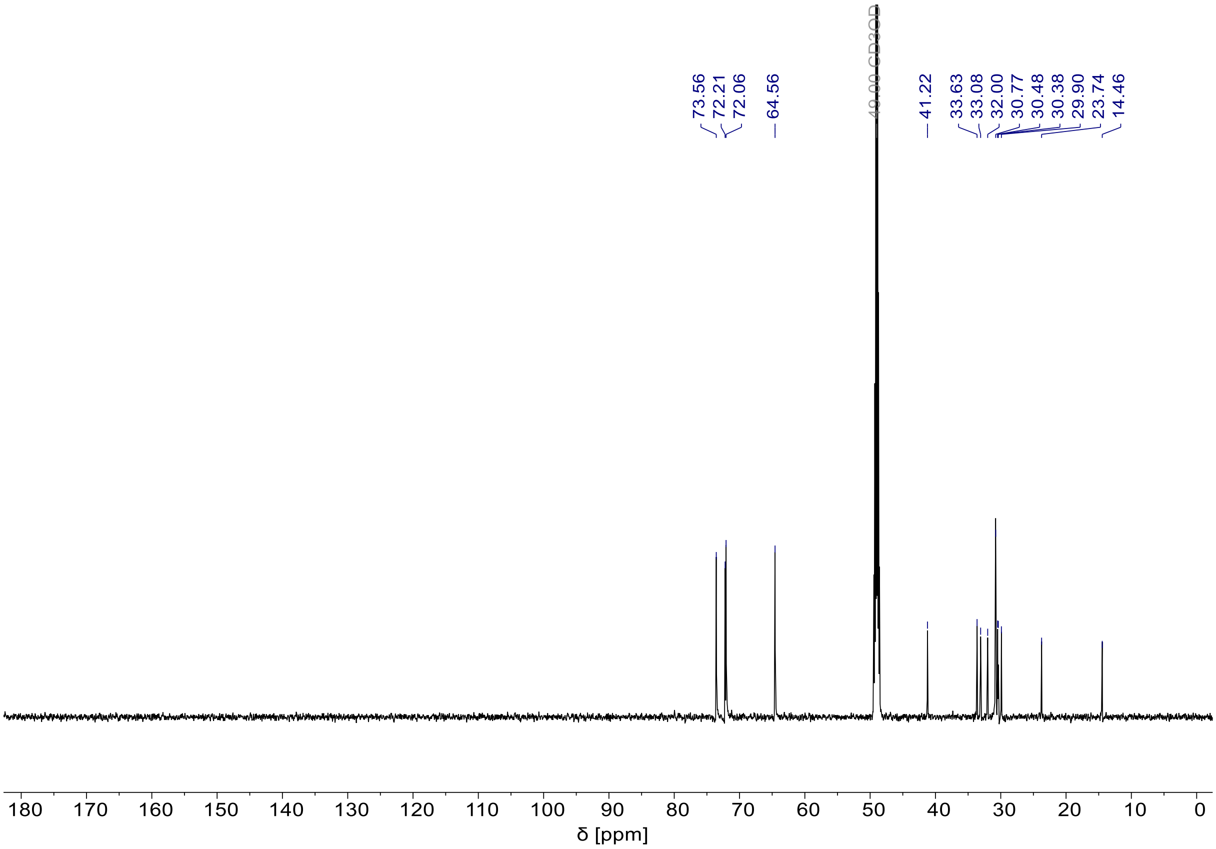

Figure S21: ^{13}C NMR (151 MHz, MeOD) of (*R*,*R*)-**dTG-C$_{12}$**.

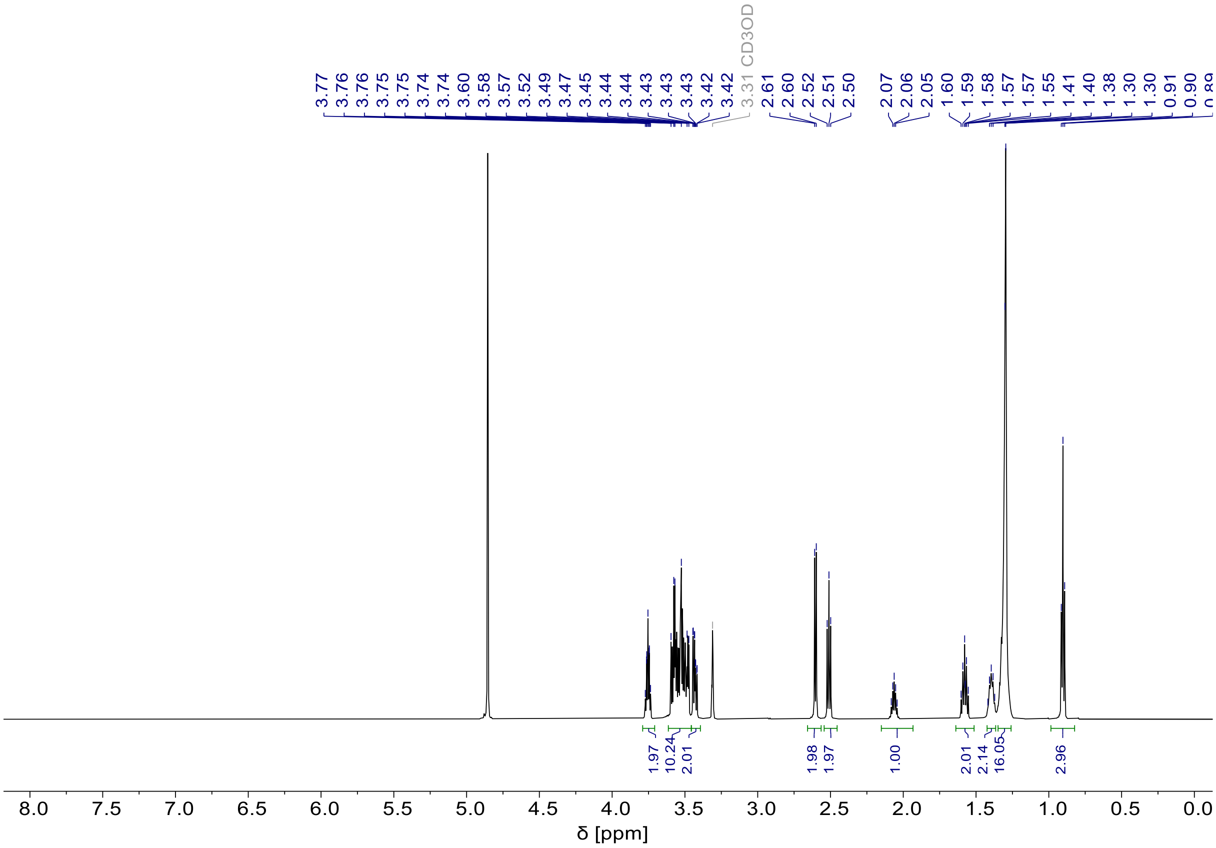

Figure S22: ^{1}H NMR (600 MHz, MeOD) of (*S*,*S*)-**dTG-C12**.

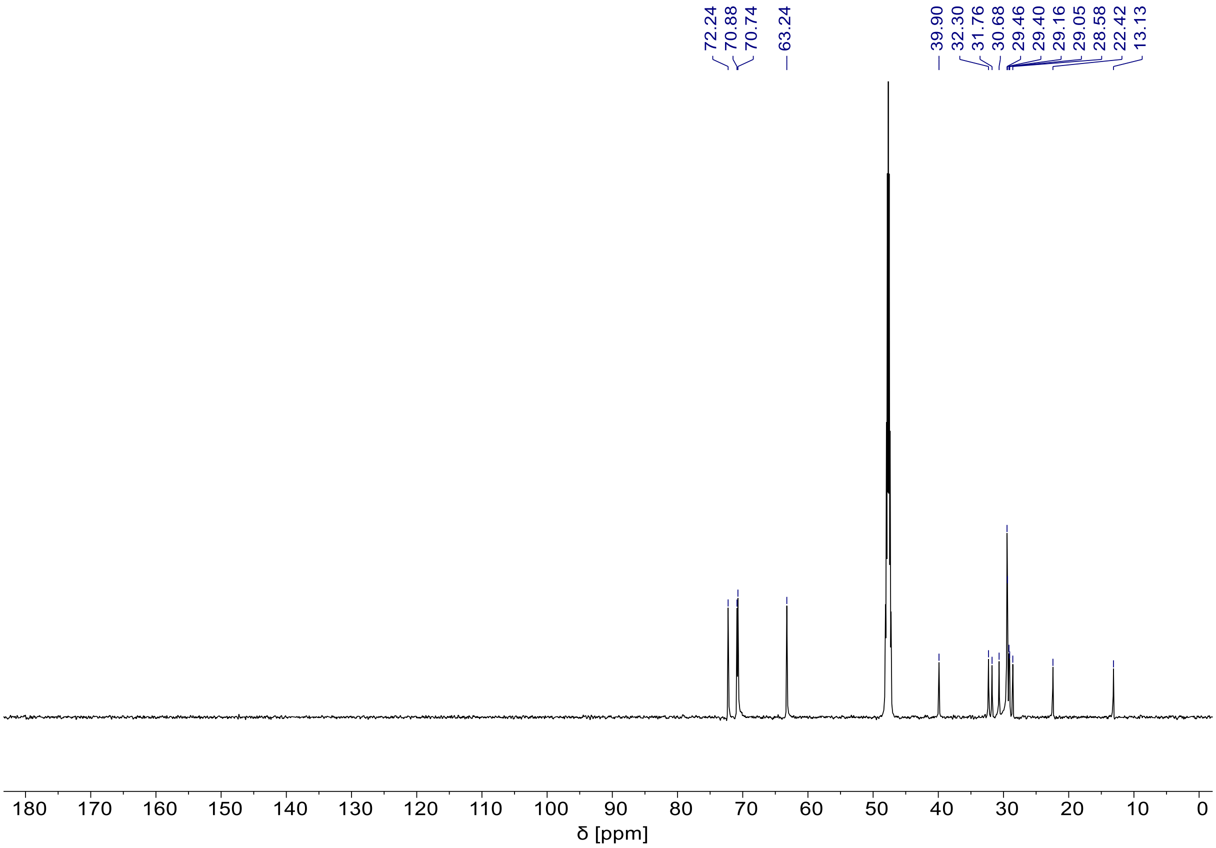

Figure S23: ^{13}C NMR (151 MHz, MeOD) of (*S*,*S*)-**dTG-C12**.

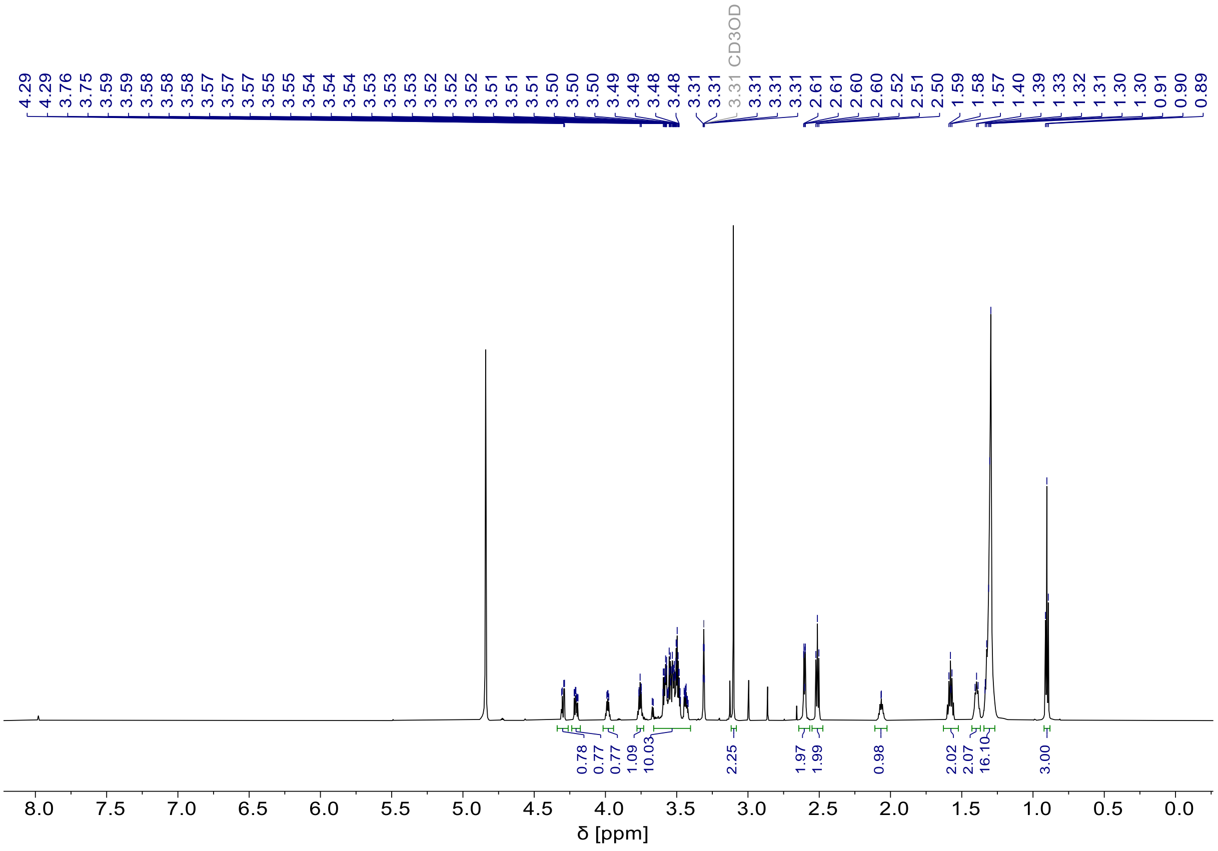

Figure S24: ^{1}H NMR (700 MHz, MeOD) of **3**.

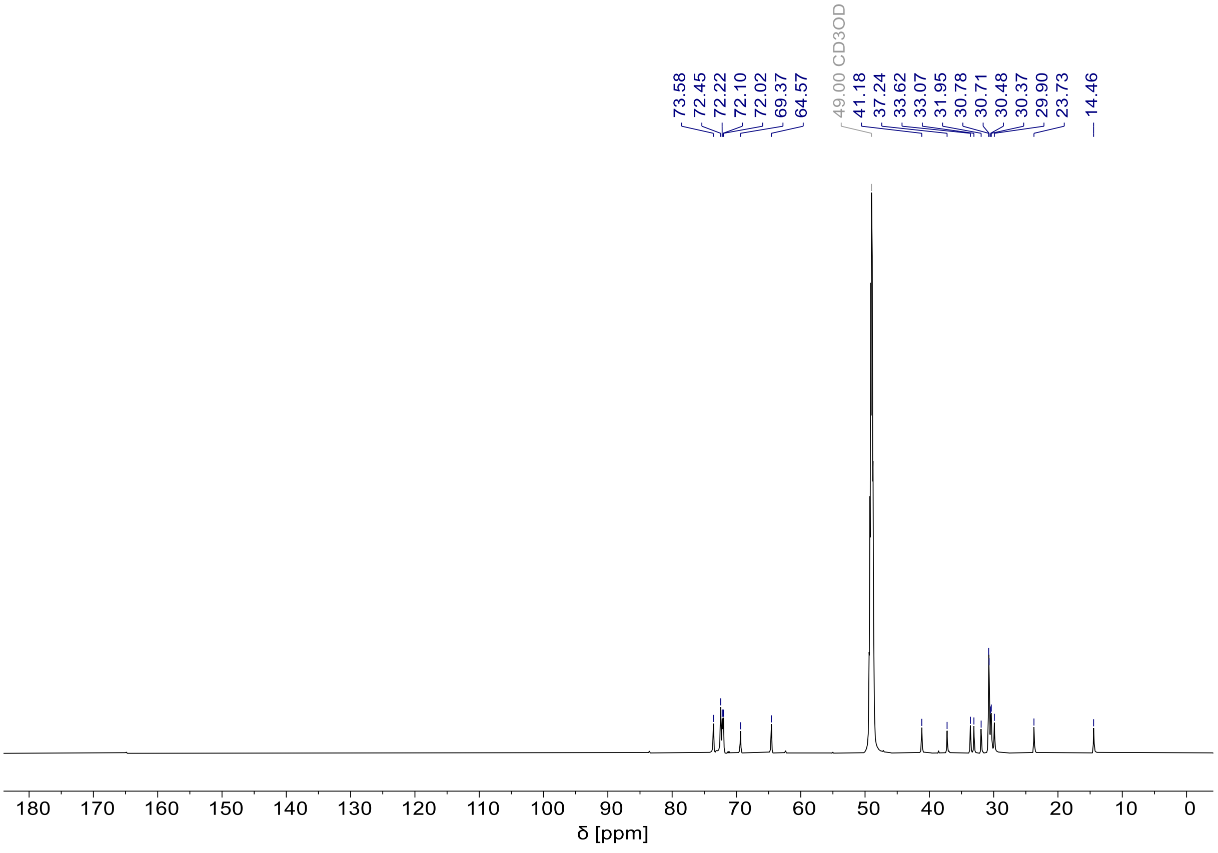

Figure S25: ^{13}C NMR (176 MHz, MeOD) of **3**.

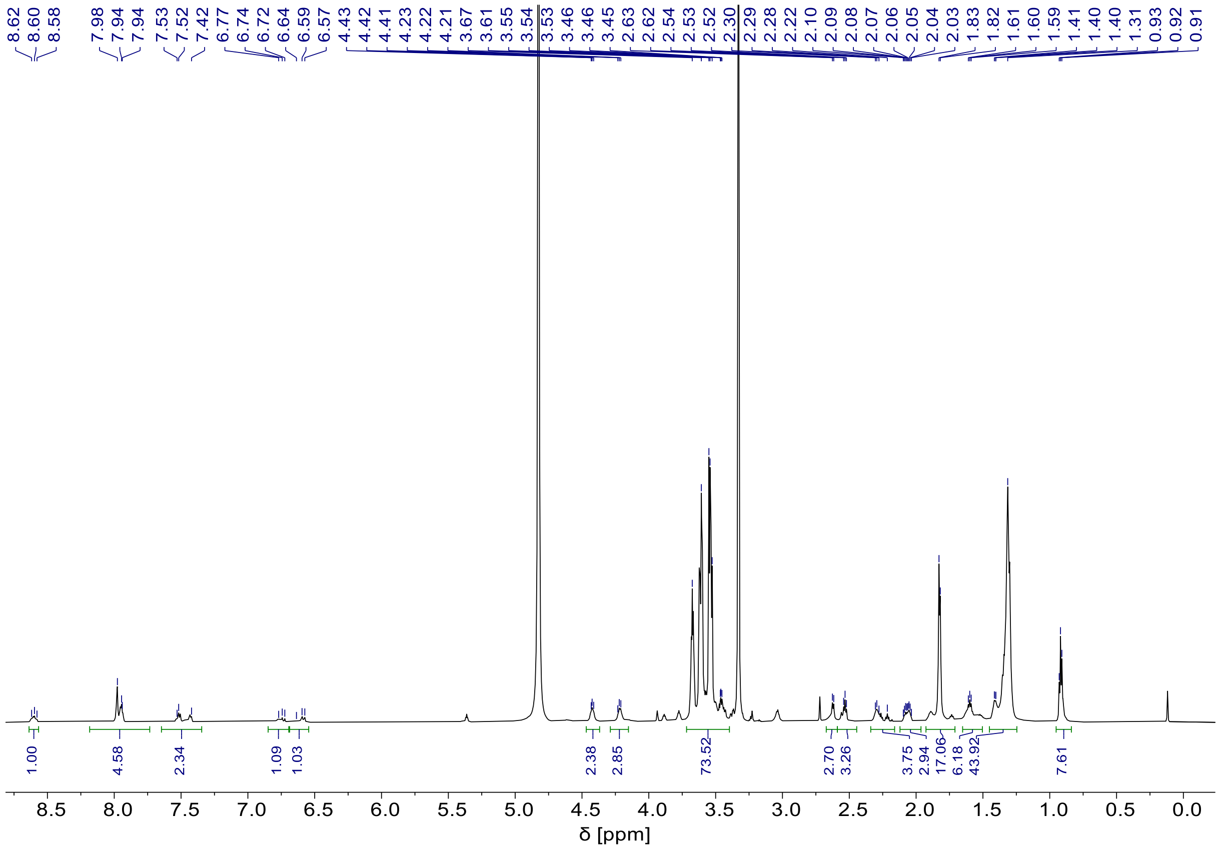

Figure S26: ^{1}H NMR (700 MHz, MeOD) of **Cy3-dTG-C$_{12}$**.
